# Supplementary material for: Quantifying the predictability of renewable energy data for improving power systems decision-making
Source: Patterns (N Y). 2023 Mar 24;4(4):100708. doi: 10.1016/j.patter.2023.100708 (PMC10140613; doi:10.1016/j.patter.2023.100708)
Supplement: Document S1. Figures S1–S5 and Note S1 [file mmc1.pdf]

Patterns, Volume *4*

Supplemental information

Quantifying the predictability of renewable energy data for improving power systems decision-making

Sahand Karimi-Arpanahi, S. Ali Pourmousavi, and Nariman Mahdavi

Note S1: Why is the predictability of PV generation not coupled with the factors currently being considered in the decision-making processes?

A crucial question regarding the importance of the proposed predictability measure for renewable generation is what additional information it can provide that current measures lack in the decision-making processes. In this respect, as the "fuel" of renewable energy sources is weather-related (e.g., solar irradiance or wind speed), a common counterargument is that the predictability of renewable energy sources is coupled with weather-related factors, which are already being considered.

To address this concern, we first acknowledge that PV generation predictability is coupled with short-term weather patterns (e.g., 1, 5 or 10-minute changes) since they are the main reason behind short-term changes in renewable generation. However, the way the weather-related factors are considered in decision-making processes does not bring into the picture the impact of these short-term changes on the predictability of renewable generation and its impacts; hence, these factors are not inherently correlated with the short-term predictability of renewable energy sources. To better explain the problem, let us focus on finding the best location for a solar farm, which typically involves the most comprehensive weather-related studies. Because of its impact on the return on investment, a detailed solar resource assessment is done by the investors to find the best location. Usually, they use earlier typical meteorological year (TMY) and satellite solar irradiance data to find one or more potential locations that would provide the highest annual yield. Then, a good practice is to install weather monitoring stations at potential locations to calibrate the satellite data and track the weather conditions over a period to improve the accuracy of yield estimation. Based on the current practices, the best location is the one that provides a high energy yield with minimal changes over the years and facilitates the safe operation of the solar farm. The former is estimated based on the solar irradiance metrics, such as monthly or annual global horizontal irradiance (GHI), direct normal irradiance (DNI), or global tilted irradiance (GTI), together with the cloud opacity or Clearness Index, in the potential solar farm locations. The safe operation is ensured based on tracking higher resolution meteorological data, such as wind gusts, rainfall, and clouds, to ensure the plant is safe from severe weather issues (such as floods), and it can operate safely during various events (for example during start-ups, shutdowns, and transients) [S1]. Therefore, if the safe operation of a plant can be ensured, the location with the highest solar power yield is typically chosen.

While affecting the decisions, none of these factors is inherently coupled with the short-term predictability of solar PV generation. Yet, the power grid's safe and efficient operation depends on the predictability of net demand and generation. That is why current (and especially the future) electricity markets reward the generation with higher predictability.

Here, we intend to reject a potential counterargument for the importance of PV generation predictability: the proposed predictability measure (WPE of dimension 6 and a

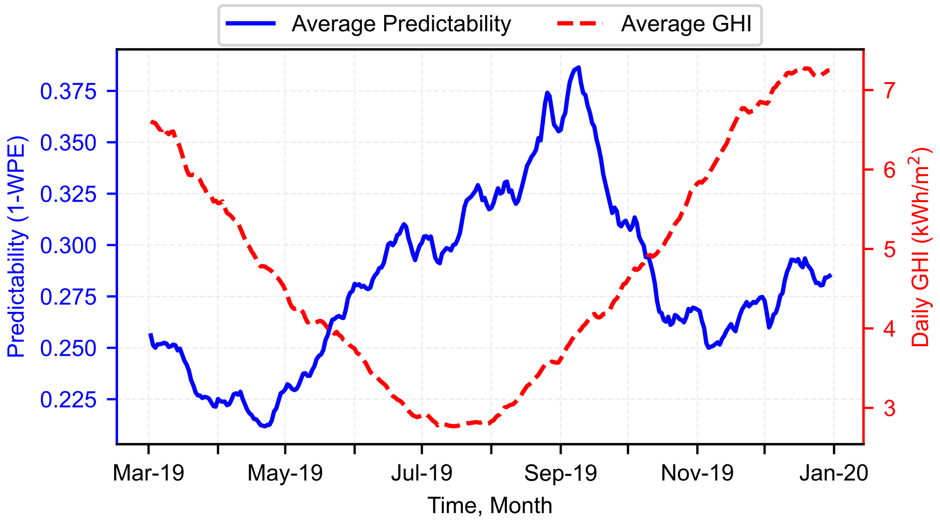

Figure S1. Negative correlation between the predictability of PV generation and the average daily GHI over time in NSW.

resampling interval of 10 minutes) is correlated with the monthly or annual solar irradiance. In other words, a location with higher all-sky solar irradiance (i.e., solar irradiance when the cloud opacity and its impact on the irradiance at the land surface is considered) would have higher generation predictability. As annual or monthly solar irradiance is the most critical factor in such decisions, if there was a link between the two, considering predictability would become unnecessary. Therefore, we have conducted the following analyses to reject this hypothesis.

Using our real-world PV generation dataset [S2], we first demonstrate that the predictability of PV generation is not coupled with solar irradiance. To do so, we compare the PV generation predictability (1-WPE) of all rooftop systems in the states of South Australia (SA) and New South Wales (NSW) over a year (by rolling two-month windows) with the average all-sky GHI in the same locations. Our rooftop PV generation dataset, respectively, includes the time series of 106 and 74 postcodes in the two states. Also, GHI data of these postcodes were obtained from the National Aeronautics and Space Administration (NASA) Langley Research Center (LaRC) Prediction of Worldwide Energy Resource (POWER) Project funded through the NASA Earth Science/Applied Science Program [S3]. Figures S1 and S2 show the average PV generation predictability and the average daily GHI over time for the states of NSW and SA, respectively. In SA, there is a positive correlation between the average GHI and the predictability over time, while in NSW, there is a negative correlation. In other words, unlike in SA, the two parameters not only show no meaningful correlation in NSW but also are negatively correlated. This means that although they can be correlated over time in some locations

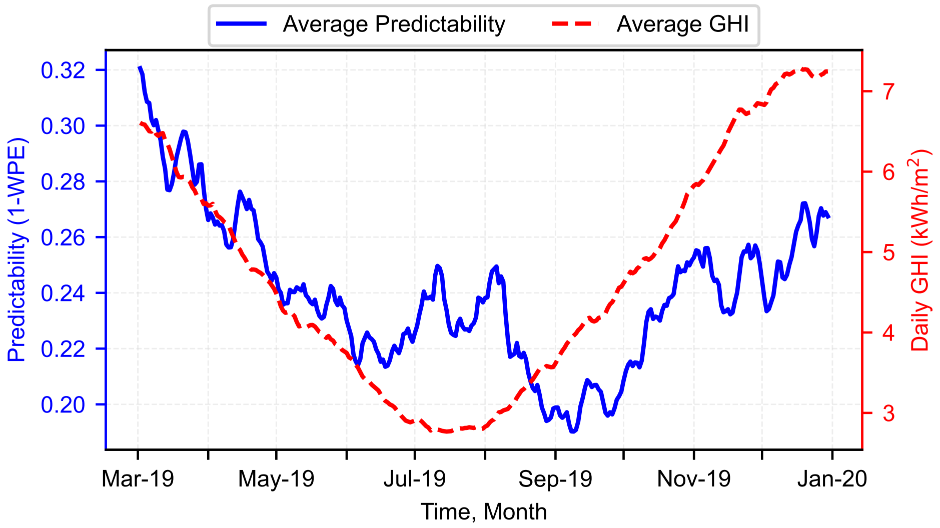

Figure S2. Positive correlation between the predictability of PV generation and the average daily GHI over time in SA.

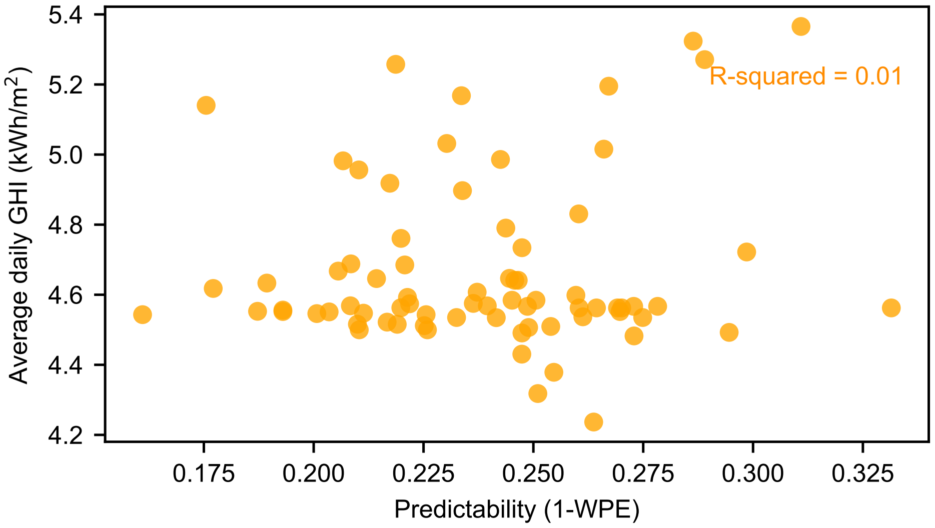

Figure S3. No correlation between the predictability of PV generation and the average daily GHI in different postcodes of NSW.

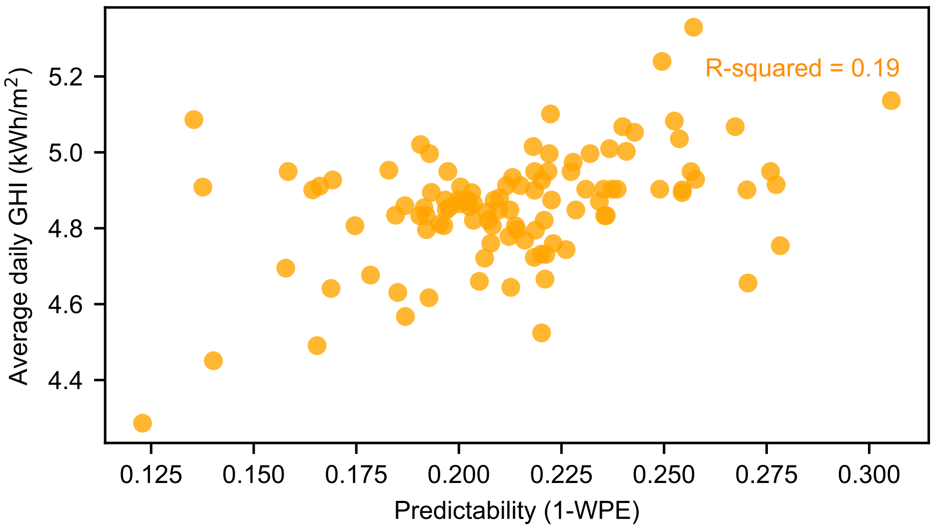

Figure S4. **Weak positive correlation between the predictability of PV generation and the average daily GHI in different postcodes of SA.**

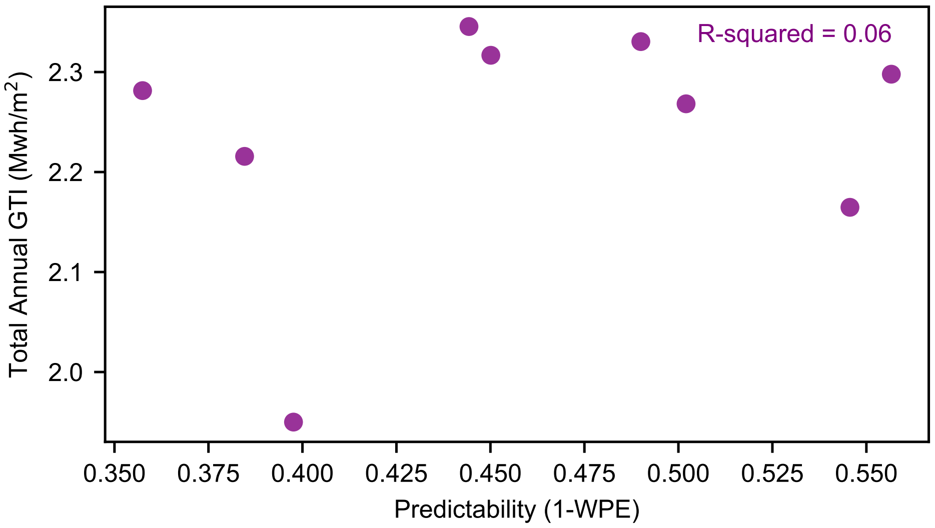

Figure S5. **No correlation between the predictability of GTI and the annual GTI in potential solar farm locations in NSW.**

(due to the weather patterns of that region), we cannot generalize the existence of the correlation to every region. This rejects the assumption that all-sky solar irradiance (or other associated factors) and PV generation predictability are inherently coupled.

Furthermore, to test the existence of any coupling between the PV generation predictability and the average daily solar irradiance of different locations, we calculated the correlation between these two factors for different postcodes in SA and in NSW, using our rooftop PV generation dataset and GHI data obtained from [S4]. As shown in Figure S3, there is no meaningful correlation between these two factors in NSW. However, doing the same analysis for SA, we found a weak correlation between these two factors, as shown in Figure S4. As previously noted, while there might be a correlation between these two factors in some locations, it is not universal; hence, they are not inherently coupled, and one cannot be used as a proxy variable for the other in decision-making processes.

Additionally, to check if the independence between predictability and average solar irradiance holds for solar farm locations in NSW (which usually have relatively high irradiance compared to urban areas), we used a dataset consisting of the 5-minute GTI time series (from August 2021 to August 2022) for single-axis sun-tracking panels in 9 potential solar farm locations in NSW. Figures 8.(a) and 8.(b) in the main manuscript, respectively, show the total annual GTI values and the predictability for these locations in NSW. We can see that the locations with higher annual GTI do not necessarily have higher predictability. For better illustration, Figure S5 shows the scatter plot of two parameters, exhibiting no correlation between them. This analysis shows that the short-term predictability of GTI (or solar irradiance) in a location is not coupled with its monthly or annual GTI, similar to the PV generation. Consequently, the location with the highest solar irradiance might not necessarily present high predictability of PV generation.

References

S1. Mehos, M. *et al.* Concentrating solar power best practices study. Tech. rep. (National Renewable Energy Lab.(NREL), Golden, CO (United States), 2020).

S2. 2019 Australian Voltage and PV Generation as part of ARENA grant ARP128. `http://www.solaranalytics.com/data`.

S3. The Prediction of Worldwide Energy Resources (POWER) Project. 2022. `https://power.larc.nasa.gov/`.

S4. Australian Hourly Solar Irradiance Gridded Data. The Bureau of Meteorology, 2015. `http://www.bom.gov.au/climate/how/IDCJAD0111.shtml`.
