# Supplementary material for: Quantifying the predictability of renewable energy data for improving power systems decision-making
Source: Patterns (N Y). 2023 Mar 24;4(4):100708. doi: 10.1016/j.patter.2023.100708 (PMC10140613; doi:10.1016/j.patter.2023.100708)
Supplement: Document S2. Article plus supplemental information [file mmc2.pdf]

Article
Patterns

Quantifying the predictability of renewable energy data for improving power systems decision-making

Graphical abstract

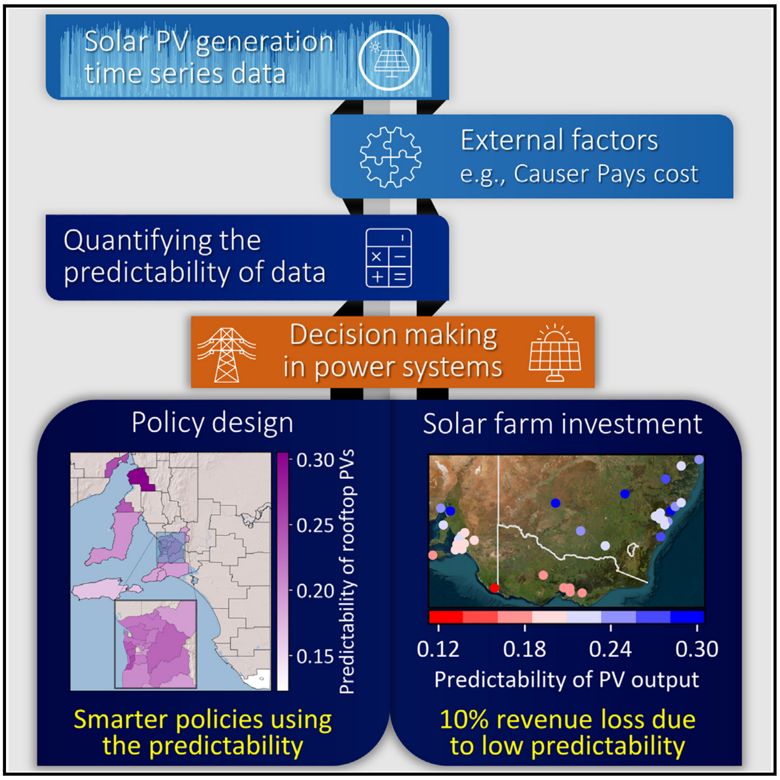

Highlights

- Effective method found to quantify the predictability of renewable generation data
- Some applications of predictability in the power systems domain discussed
- Low generation predictability can reduce up to 10% of solar farms' revenue
- Considering predictability in policy design imperative for a reliable power system

Authors

Sahand Karimi-Arpanahi,
S. Ali Pourmousavi, Nariman Mahdavi

Correspondence

sahand.karimi-arpanahi@adelaide.edu.au

In brief

The net-zero energy transition requires more renewable energy in the electricity generation mix. However, these sources have unpredictable generation that poses challenges for the power grid and lowers profits for investors. Currently there is no reliable method for quantifying renewable energy predictability. This paper presents a predictability measure and demonstrates its significance for the energy industry. Our findings indicate that considering predictability in decision-making leads to better policies, higher profits for investors, and lower electricity costs for customers.

Karimi-Arpanahi et al., 2023, Patterns *4*, 100708
April 14, 2023 © 2023 The Author(s).
https://doi.org/10.1016/j.patter.2023.100708
CellPress

Patterns

CellPress
OPEN ACCESS

Article

Quantifying the predictability of renewable energy data for improving power systems decision-making

Sahand Karimi-Arpanahi,[1,2,3,*] S. Ali Pourmousavi,[1] and Nariman Mahdavi[2]
[1]School of Electrical and Mechanical Engineering, University of Adelaide, Adelaide, SA 5005, Australia
[2]CSIRO Energy, Newcastle, NSW 2304, Australia
[3]Lead contact
*Correspondence: sahand.karimi-arpanahi@adelaide.edu.au
https://doi.org/10.1016/j.patter.2023.100708

THE BIGGER PICTURE As energy generation from solar and wind sources is subject to weather variability, short- and long-term decisions in the electricity sector are heavily influenced by their generation forecasts. Consequently, millions of dollars of public and private funds have been spent on developing accurate forecasting tools. In this article we argue that, while these tools are essential, there is a complementary aspect to this problem, namely the inherently limited predictability of renewable energy data. Researchers and practitioners in the energy sector have often overlooked this aspect. Here, we present a reliable method for quantifying the data predictability of renewable energy sources and illustrate its applications through real-world examples, from private investment decisions to public policies. Our findings indicate that considering predictability in power system decisions can save millions of dollars in operation costs, prevent the waste of clean energy, and lower electricity costs.

1 2 3 4 5 **Development/Pre-production:** Data science output has been rolled out/validated across multiple domains/problems

SUMMARY

Decision-making in the power systems domain often relies on predictions of renewable generation. While sophisticated forecasting methods have been developed to improve the accuracy of such predictions, their accuracy is limited by the inherent predictability of the data used. However, the predictability of time series data cannot be measured by existing prediction techniques. This important measure has been overlooked by researchers and practitioners in the power systems domain. In this paper, we systematically assess the suitability of various predictability measures for renewable generation time series data, revealing the best method and providing instructions for tuning it. Using real-world examples, we then illustrate how predictability could save end users and investors millions of dollars in the electricity sector.

INTRODUCTION

In 2023, renewable electricity generation is expected to increase by more than 9%, surpassing 9,300 TWh worldwide.[1] Two-thirds of this growth comes from the increase in solar photovoltaic (PV) and wind energy generation, demonstrating their crucial role in reducing greenhouse gas (GHG) emissions.[1] A surge in the new solar and wind farm installation is anticipated in the coming years with the recent commitment of more than 40 countries to phase out coal-fired power plants at the COP26 climate summit.[2] Despite the evident environmental and economic benefits of PV and wind generation sources, their output is intermittent and highly uncertain and, hence, undispatchable (the dispatchability of an electricity generation source means that their output power can be adjusted within the physical limits of the generator based on the electricity grid requirements).[3] This, in turn, can cause frequent mismatches between electricity supply and demand in power grids, which affects the planning and design of power systems, electricity market operation, and several other aspects of power systems that depend on dispatchability and accurate prediction of electricity generation.[4] As a result, renewable generation prediction has become an integral part of numerous decision-making processes related to power systems.

These decisions, made by different stakeholders in electricity systems, are usually split into short- and long-term decisions.

Check for updates

This is an open access article under the CC BY license (http://creativecommons.org/licenses/by/4.0/).

Short-term decisions are typically operational in nature by focusing on how to make the best use of resources in the short term, e.g., day-ahead and real-time electricity markets, in which power plants and transmission system operation will be scheduled. In contrast, long-term decisions, made when considering future achievements, lead decision-makers to take actions different from those they would usually do. GHG emission reduction through renewable energy policy design is a classic example of a long-term decision typically made by governments. Other examples of long-term decisions are (1) shaping the long-term strategies of renewable energy in a jurisdiction based on solar and wind atlases[5–8] or (2) a private investor who decides among multiple energy projects by looking at the potential yield of the sites in several decades ahead.[9] A short-term example is the energy procurement by the power system operators in the day-ahead or real-time electricity markets, where they use day- or several-minutes-ahead predictions of renewable generation for making least-cost, reliable decisions.[10] Whether short- or long-term decisions, some form of renewable generation prediction is involved in the decision-making process. Thus, one can realize the sheer importance of renewable generation prediction in the electricity industry.

In recent years, much work has been done on developing short-term forecasting methods (prediction horizons of a few minutes to a day) for wind and solar PV generation.[11–13] These scientific efforts have been supported by generous grant money, mostly from government funding agencies,[14,15] hoping to improve the forecasting accuracy of solar or wind generation. In addition to academic efforts, numerous companies[16–18] are commercially providing renewable generation forecast services for electricity market participants and operators. Nonetheless, all the existing forecasting methods in the literature use historical data directly or indirectly for prediction, even those using numerical weather forecast models. As a result, regardless of the type, granularity, and prediction horizon of forecasting methods, their accuracy is restricted by inherent predictability of the historical data. Predictability in this context means "the ability to determine ahead of time the availability of a generation resource,"[4] such as wind and solar energy. Despite the importance of inherent predictability in this context, most studies that explored the uncertainty in renewable generation relied solely on forecasting methods to evaluate their impacts on electricity systems.[11–13] There are a few studies on the predictability of wind speed or power generation for some power systems applications, where prediction error metrics were used as a predictability measure.[19–21] While lower forecast errors could imply higher predictability in a given time series, they cannot be used as a surrogate parameter for the predictability because of the following issues.

1. Which forecasting method? Each prediction technique uses a predefined model, e.g., linear or nonlinear, or deterministic or stochastic, to learn the existing patterns in a time series.[22] Therefore, their predictions do not reflect the inherent predictability of the time series.[23,24] Even if we could know the characteristics of the underlying data, there would have been numerous prediction techniques for each class of problems with potentially different performance and accuracy, making it arduous to justify using a specific forecasting method. The other way is to try all those methods which may be impossible or inefficient.
2. What prediction horizon? The performance of forecasting methods can vary significantly by the prediction horizon and highly depends on seasonality and the time of day.[25,26] Therefore, a particular prediction horizon should be picked and justified, which is challenging.
3. Which prediction error metric? There are many prediction error metrics (mainly categorized into scale-dependent, percentage, and scaled errors) to evaluate the performance of a forecasting method, each with specific limitations.[27] Choosing the proper prediction error metric depends on the data and their quality, the loss function of the forecasting method, goals, and applications. Thus, another arduous task is to choose and justify an error metric representing the predictability for a given time series.

Therefore, using a proper predictability theory that does not involve the above issues is necessary.

The concept of time series predictability has been investigated in other disciplines, such as climatology,[28] ecology,[29] epidemiology,[30] financial markets,[31] and communication systems,[32] to mention a few. In power system studies, however, predictability measures have rarely been used despite their multifaceted applications.[26,33,34] Consequently, quantifying the predictability of renewable generation has been entirely overlooked in practical decision-making processes and relevant research studies where power systems are concerned.

This article investigates the suitability of various potential predictability measures used in other disciplines for the renewable generation time series. After finding the best predictability measure, we tune its hyperparameters to suit our application. Then, through studying different real-world examples, we demonstrate that considering the predictability of renewable generation as a deciding factor is essential for many long-term decisions in electric power systems. We argue that while renewable generation forecasting methods are indeed necessary, incorporating the inherent predictability of renewable generation in long-term decision-making processes can help achieve better decisions. In this regard, we demonstrate that solar farms' expected profit strongly correlates with their generation predictability. Then, by revealing the strong dependency of predictability on the location of solar farms, we establish that these decisions will be suboptimal unless predictability is considered in the investment decisions of the renewable plants. Also, by presenting the findings from our study on the rooftop PV systems across South Australia (SA), Australia, we illustrate that ignoring the predictability in renewable energy policymaking has led to more unpredictable generation in the power grid, potentially resulting in higher reserve market prices and green energy spillage. Lastly, by showing the strong relationship between predictability and the location of solar PV systems, we show how this feature can help public service sectors to achieve better solutions for managing a power system with high renewable energy integration. These findings highlight the crucial role of quantifying renewable generation predictability in making effective long-term decisions related to power systems.

CellPress
OPEN ACCESS

Table 1. Summary of the assessed predictability measures

Predictability measure	Introduced in	No. of hyperparameters	Applied in
Dispersion entropy (DE)	2016 (Rostaghi and Azami[45])	2 (dimension, class)	mechanics (Rostaghi et al.[81]), biomedical engineering (Azami et al.[82])
Permutation entropy (PE)	2002 (Bandt and Pompe[37])	1 (dimension)	medicine (Li et al.[83]), epidemiology (Scarpino et al.[30]), economics (Zumino et al.[84])
Sample entropy (SaE)	2000 (Richman and Moorman[36])	2 (dimension, match criterion)	biology (Kaffashi et al.[85]), neurology (Li et al.[86]), biomedical engineering (Yentes et al.[87])
Spectral entropy (SpE)	1991 (Inouye et al.[76])	0	economics (Georg[88]), neurology (Li et al.[89])
Weighted PE (WPE)	2013 (Fadlallah et al.[43])	1 (dimension)	ecology (Pennekamp et al.[29]), computer science (Garland et al.[24]), climatology (Huang and Fu[28])

RESULTS

Finding a universal predictability measure

The predictability of a dynamical system reflects the extent to which its future state can be anticipated. In a system with high predictability, the future state of the system can be accurately predicted using previously known states. For example, periodic or constant signals fall into this class. Conversely, a dynamical system with low predictability refers to a system whose future state cannot be determined among various possibilities. White noise, for example, falls into this category, where the historical data give no insight into the future, regardless of the forecasting method. On this basis, predictability is an inherent property of dynamical systems.[23]

As there is little consensus on the definition of the signal's predictability, various measures have been developed and used to determine the predictability of time series data. Most of these measures can be categorized into two groups: (information) entropy-based metrics and fractal theory-based metrics. While the former group is more widely known for measuring the predictability of time series,[35–37] the metrics from the fractal theory, specifically the self-similarity parameter (also known as Hurst exponent), have been used to represent the predictive structure of a signal.[26,32,38,39] Nonetheless these two groups are fundamentally different, as each captures a particular aspect of the time series predictability. In information theory, entropy is used to characterize the complexity or compressibility of data.[40,41] Data with high entropy include minimal redundant information, making them difficult to compress. In other words, these data have a high level of randomness or unpredictability, making it difficult to identify patterns or regularities that can be exploited to compress the data. In contrast, data with low entropy tend to be easier to compress as they have a higher level of regularity and predictability. In fractal theory, however, the Hurst exponent shows the degree of self-similarity and long-range dependence of a signal.[32] In this context, higher long-range dependence means that past values can give a better insight into the future. Nevertheless, as the Hurst exponent of a time series cannot be directly calculated, various methods have been developed to estimate it, each of which would provide a different value.[38] It is, therefore, challenging to establish a reliable and robust measure of predictability based on the Hurst exponent. On the contrary, the entropy-based metrics developed over the past three decades can be readily calculated using efficient, reliable methods. Some examples of such metrics are approximate entropy (AE),[35] sample entropy (SaE),[36] and permutation entropy (PE).[37] They are also closely related to the known predictability of dynamical systems, quantified by Lyapunov exponents and Kolmogorov-Sinai entropy.[24,36,37] These features (i.e., direct representation of uncertainty and reliable calculation methods) make entropy-based metrics a better way to quantify the predictability of renewable generation data.

Various entropy-based metrics have been introduced in different disciplines to measure complexity or predictability, where a time series with higher entropy is considered more complex or less predictable, and vice versa. Here, we investigate which method, among the prominent approaches, is the most effective for measuring the predictability of renewable generation time series. Table 1 provides a summary of these entropy-based metrics. In the experimental procedures section, we describe how these measures are calculated.

To assess the suitability of the entropy-based metrics introduced in Table 1, we used a dataset consisting of 1,000 rooftop PV generation time series belonging to Australian households known by their postcodes.[42] The data are available for 1 year, from January 1, 2019 to January 1, 2020, with a sampling interval of 5 min. The dataset was checked and cleansed before analysis, where time series with more than 200 missing values and significant generation clipping (if a PV system has the same maximum generation for 7 months, we consider significant generation clipping has happened, possibly due to the rooftop PV export limits in Australia) were removed from the dataset. This is important because if the renewable generation time series is significantly affected by external factors, such as clipping and self-curtailment, the patterns of the affected time series would no longer represent the inherent features of the renewable generation, thus being unsuitable for measuring the inherent predictability of a renewable energy source in a given time and location. After data cleaning, 335 reliable time series remained to analyze the generation of PV systems in SA, New South Wales (NSW), and Victoria (VIC).

In the first step, we carried out a preliminary assessment of the potential predictability measures. Using the five different entropy-based metrics, we compared the entropy values of three randomly selected PV generation time series from our dataset with white Gaussian noise (WGN) and a pure sine wave signal

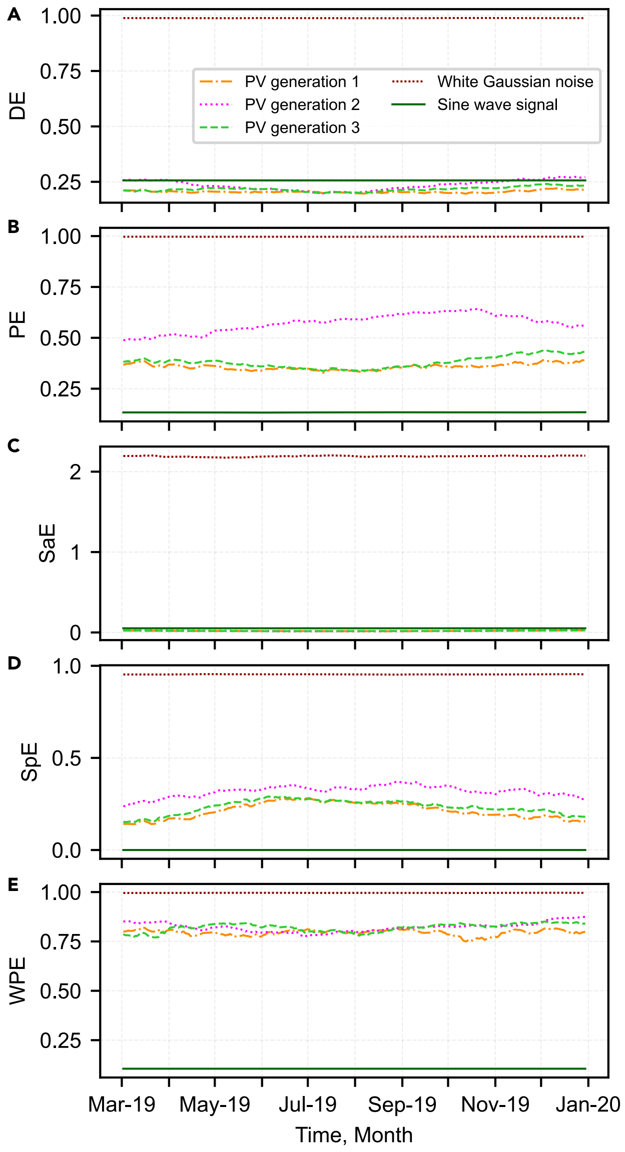

Figure 1. Preliminary assessment of the suitability of potential predictability measures for renewable generation time series

(A), (B), (C), (D), and (E), respectively, show the values over time obtained by dispersion entropy (DE), permutation entropy (PE), sample entropy (SaE), spectral entropy (SpE), and weighted permutation entropy (WPE) for a sine wave signal (with a 1-day cycle), a white Gaussian noise (WGN), and three randomly selected PV generation time series from our dataset. These are calculated using 2-month rolling windows that move 1 day forward at a time. The hyperparameters are set to the commonly used values of each metric (i.e., the dimension of PE and WPE is 6, both the class and dimension of DE are 5, and the dimension of SE is 3).

over 1 year using 2-month rolling windows. In this study, WGN, a pure random noise, is expected to show high entropy values (i.e., low predictability) compared with other time series. At the same time the sine wave, a perfectly periodic signal, must have the lowest entropy (i.e., highest predictability). Also, the three PV generation time series are expected to have entropy values between the two extreme cases. As shown in Figure 1, WGN and sine wave signals consistently indicate the highest and lowest entropy values, respectively, over the course of a year when PE, weighted PE (WPE), and spectral entropy (SpE) are used as the predictability measures. Dispersion entropy (DE) and SaE, however, fail the preliminary test because they incorrectly indicate that the sine wave signal is less predictable than all three PV generation time series. While the entropy values of each metric will differ based on the hyperparameters used, the qualitative results will remain the same, i.e., DE and SaE fail the test regardless. Thus, they are unsuitable metrics for measuring the predictability of PV generation time series, and the most appropriate metric should be chosen from SpE, PE, or WPE.

Additionally, we should determine the best hyperparameters and resampling intervals such that the predictability measure can capture the most important predictive structures in the renewable generation time series. While SpE calculation does not include any hyperparameter, both PE and WPE values are calculated based on a hyperparameter (i.e., embedding dimension) that can take any integer from 3 to 7.[37,43] However, as the maximum embedding dimension is restricted by the length of the time series,[44] our options would be limited to from 3 to 6. Resampling the original 5-min data is also necessary for measuring the inherent predictability of renewable generation because the mentioned entropy-based metrics, i.e., PE, SaE, WPE, and DE, are measured on the basis of patterns in the vectors established from the 2 to 8 sequential data points in the time series.[36,37,43,45] The finite length of the vectors may thus lead to neglecting existing predictive structures in the vectors with higher lengths. Conversely, creating longer vectors by choosing a longer resampling interval may lead to disregard of predictive patterns in the data, as the higher-resolution dynamics will disappear in resampling. Hence, finding the optimal resampling interval is necessary for measuring predictability.

To find the best metric, hyperparameters, and resampling intervals, we calculated the entropy values of all PV generation time series in our dataset, using the candidate predictability measures with different hyperparameters and resampling intervals. The best measure was chosen by comparing the correlation between the entropy values and the average prediction errors of all time series in our dataset. No matter what prediction method, horizon, or error metric is used, a time series with low entropy (i.e., high predictability) should exhibit relatively low prediction errors. To this end, we compared different predictability measures based on their average correlation with 16 sets of prediction errors consisting of four prediction horizons (i.e., 5 min, 10 min, 15 min, and 20 min ahead), two forecasting methods (i.e., autoregressive integrated moving average [ARIMA] and naive), and two error metrics (i.e., the normalized absolute error [NMAE] and the normalized root-mean-square error [NRMSE]). The maximum prediction horizon was 20 min, the longest horizon relevant to renewable generation prediction in the Australian electricity market (while our case studies are limited to the Australian electricity market, the predictability measurement process, and the conclusions, are not). In this spot energy market, generators typically submit their final bids (the generation forecasts in the case of renewable plants) for each dispatch target 5–10 min ahead. However, depending on the forecasting method or the operational requirements of the renewable plants, forecasts with longer horizons, i.e., 10–20 min, can be important too. We also chose different error metrics and forecasting

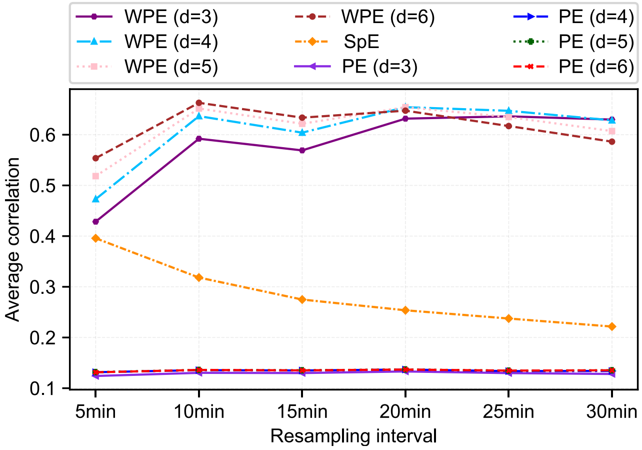

Figure 2. Finding the best predictability measure and its hyperparameters for renewable generation time series

Each line shows the average correlation between the predictability values obtained by a measure with a specific hyperparameter and 16 datasets of prediction errors, resulting from four prediction horizons (5 min, 10 min, 15 min, and 20 min ahead), two forecasting methods (ARIMA and naive), and two error metrics (NMAE and NRMSE).

methods with different learning procedures to ensure that the best metric would be selected regardless of the method and error metric. The NMAE and NRMSE of each time series for each forecasting method were calculated based on 105,080 predictions obtained for 5-, 10-, 15-, and 20-min-ahead horizons with 3 h of training data. To create the set of predictions for each time series, we used rolling windows throughout the year with one interval moving forward at a time. Finally, 16 forecast error sets were created from different combinations of prediction horizons, methods, and error metrics to find the best predictability measure for our application.

The result of this comparison is shown in Figure 2. According to the graph, WPE outperforms SpE and PE regardless of the selected hyperparameter and resampling interval. Also, increasing the resampling interval impacts the average correlation of each metric differently. Even choosing different hyperparameters for the WPE leads to different best resampling intervals (i.e., the resampling interval with the highest average correlation). Furthermore, when the resampling interval varies between 10 and 25 min, the WPE values obtained for dimensions 4–6 have an average correlation of more than 0.6 with prediction errors. This shows the robustness of the WPE measure in this application, although tuning the WPE hyperparameters would result in a better measure. Overall, the WPE with a dimension of 6 and a resampling interval of 10 min has the highest correlation among all cases, which makes it the most suitable predictability measure in this application. Figure 3 depicts the relationship between the tuned WPE and the NMAE and NRMSE values of the time series obtained for the two forecasting methods and the four prediction horizons. All 16 scatterplots show a statistically significant correlation between the WPE and the prediction error. The R^2 values, shown in Figure 3, are high, given the amount of explainable short-term variability in the rooftop PV generation data. This further supports the selection of the WPE for measuring the predictability of renewable generation.

In addition to the minutes-ahead bidding in the Australian electricity market, generators should submit day-ahead generation bids. While they are not financially binding as generators can change the bids in the real-time market, they act as an advisory measure for the market operator. Therefore, an accurate day-ahead renewable generation prediction can help the operator run the market more efficiently. In this regard, Figure 4 depicts a moderate to strong correlation between the tuned WPE and the day-ahead prediction errors. We used two forecasting methods, seasonal naive and random forest, with 9 (note the qualitative results have been the same with any other training set sizes between 6 and 14 days) days for training and 1 day for testing.[27] The NRMSEs and NMAEs were obtained for a 10-day rolling window (9 days for training and 1 day for testing) throughout a year with 1 day moving forward (i.e., 354 prediction windows). Finally, the median of the NRMSEs and NMAEs was calculated for each time series in the dataset. The strong positive correlation between the day-ahead prediction errors and the WPE shows its suitability as a predictability measure for renewable generation. Since the WPE is positively correlated with prediction errors, we define the predictability index as “1 − WPE,” where a lower WPE of a given time series shows higher predictability.

Applications of predictability in power systems

Private investment decisions

Many research studies have been carried out in recent years proposing methods for finding the best location for building solar PV and wind farms,[46–49] wherein various tools and factors are used, such as solar or wind atlases, geographic information system data, transmission lines, roads, and so forth. Similar factors, in addition to some practical considerations, are taken into account by industry and investors for renewable plant investments.[50,51] For example, in Australia, marginal loss factor assessment at a potential location is a key step in building new power plants because it can greatly affect revenue and is one of the main factors considered by investors.[52] Such assessments are critical because these factors affect not only the energy yield of solar or wind farms over their lifetime but also their participation in the day-ahead and/or real-time (or spot) electricity markets, where they must meet their energy commitments. Similar to conventional power plants, solar and wind farms are penalized for deviating from their market commitments. The penalty could be particularly devastating when renewable power plants participate in a day-ahead market compared with a real-time market because of higher prediction errors in the day-ahead market (many renewable plants bid conservatively, i.e., below their actual prediction, to dodge the imbalance penalties, which results in green and cheap energy spillage). Even in a real-time market where participants can bid only a few minutes to hours before every dispatch interval, prediction errors of renewable generation can lead to significant financial penalties. For example, in the Australian National Electricity Market (NEM), the operator utilizes regulation Frequency Control Ancillary Services (FCAS) to ensure the balance between supply and demand, where the source of imbalance could be the difference between the actual production level of renewable plants and their commitment due to prediction errors. The Australian Energy Market Operator (AEMO) then recovers the

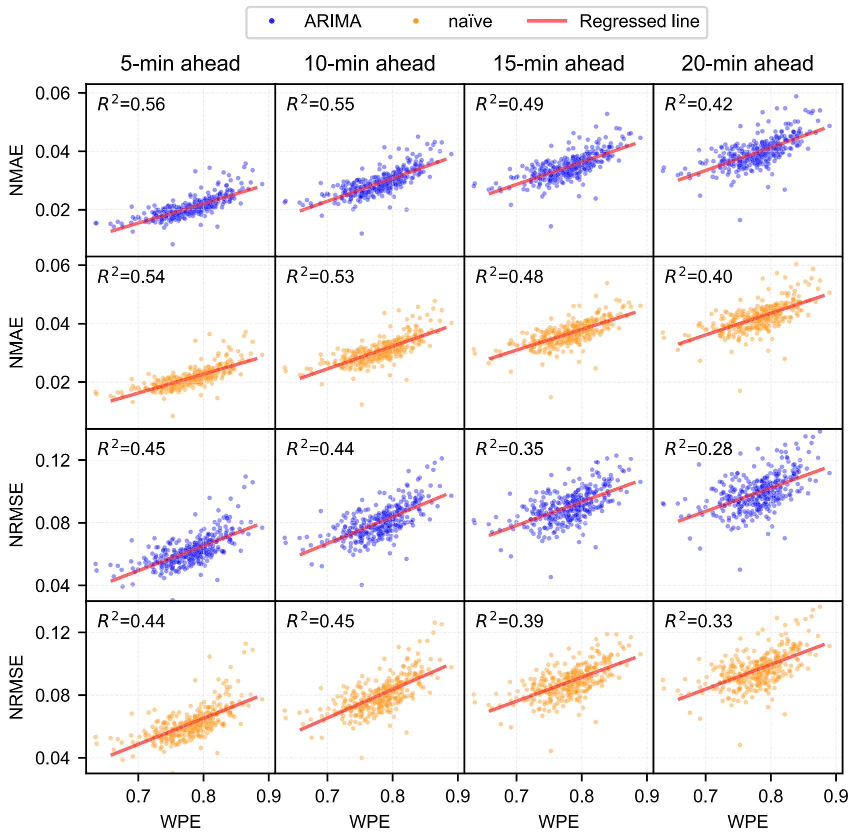

Figure 3. High correlation between the WPE and the minutes-ahead prediction errors of solar PV generation

The scatterplots show the relationship between the WPE of dimension 6 and the 10-min resampling interval, and the NRMSE and the NMAE of the PV generation data when ARIMA and naive forecasting methods are used to predict 5-, 10-, 15-, and 20-min-ahead generation.

regulation FCAS costs from market participants, including wind and solar farms, by determining how much each has contributed to the need for this service, called the "causer pays" procedure.[53] Figure 5 shows the share of regulation FCAS charges per terawatt-hour of produced energy for the NEM power plants based on fuel types in each quarter of 2020. While the total production (hence revenue) of solar PV and wind farms was much less than coal- and gas-fired plants, the share of regulation FCAS charges for the renewable plants was considerably higher than those of conventional power plants. This results in a significant reduction in the renewable plants' profit. For example, owing to such charges, four renewable plants in SA lost more than 20% of their energy market revenue in 2020.[54,55] One main reason for higher FCAS charges of solar farms is the significant prediction errors in their generation forecasts that lead to higher causer pays factors (CPFs), based on which a specific percentage of the regulation FCAS costs is assigned to each power plant.[56] Figure 6 depicts the relationship over time between the CPFs of six solar farms in NSW, gathered from AEMO,[57] and the predictability of PV generation, determined on the basis of two different datasets: (1) our rooftop PV generation dataset and (2) 5-min historical data of all-sky global tilted irradiance (GTI) at the six solar farm locations in 2019, downloaded from SolCast.[58] The figures demonstrate a strong negative correlation between the average CPF and the predictability for the actual solar PV generation and the GTI of solar farms. Each participant's monthly CPF determines their regulation FCAS market costs, so the lower the CPF, the lower the regulation FCAS charges. This further validates the choice of the WPE as the predictability measure, since the regulation market costs of solar farms, which depend on their forecasting accuracy, strongly correlate with the WPE.

To understand the impact of considering PV generation predictability on the decision to build a solar farm, we conducted a case study of nine potential locations in NSW, shown in Figure 8, to determine the best location for a hypothetical 51.8 MW solar farm. We considered two different scenarios. In the first scenario, the projected revenue of the solar farm was calculated only on the basis of the total solar energy yield, which is known as the main factor in choosing the location of a solar farm in practice. In the second scenario, we considered the revenue and the FCAS market charges caused by the unpredictability of generation in calculating the projected net revenue. Note that the solar farm capacity equals the average capacity of the six solar farms studied in the previous analysis. Also, all potential locations are situated close to transmission lines and main roads in NSW. To quantify the monetary value of PV generation predictability, we first estimated a quantitative relationship between the predictability and the CPF. The scatterplots in Figure 7A illustrate the relationship between the average predictability and the CPF of solar farms over 2-month rolling windows in NSW, based on the previous analysis shown in Figure 6. We further validated the results by depicting the relationship between the annual predictability of solar farms' GTI data and their average CPF for a year (Figure 7B). Despite the limited number of data points, in all three cases shown in the scatterplots the Pearson correlation was statistically significant, indicating a strong negative correlation between the predictability (i.e., 1 − WPE) and the CPF. Even though the predictability values of the GTI dataset are consistently higher than those of the rooftop PV system, the slope of the regressed lines are relatively similar, suggesting that a robust relationship exists between the CPF and the predictability. (This is due to many reasons. For example, PV system malfunction, panel degradation, shading, and so forth do not impact the GTI data, which leads to its higher predictability.) Finally, to compare the net revenue of the solar farm in different locations for the two scenarios, we used the GTI sun-tracking data of the locations shown in Figure 8 from August 2021 to August 2022. We set location 1, where the White Rock solar farm is installed, as our baseline in the comparison. Based on the real historical data, the White Rock solar farm had an annual

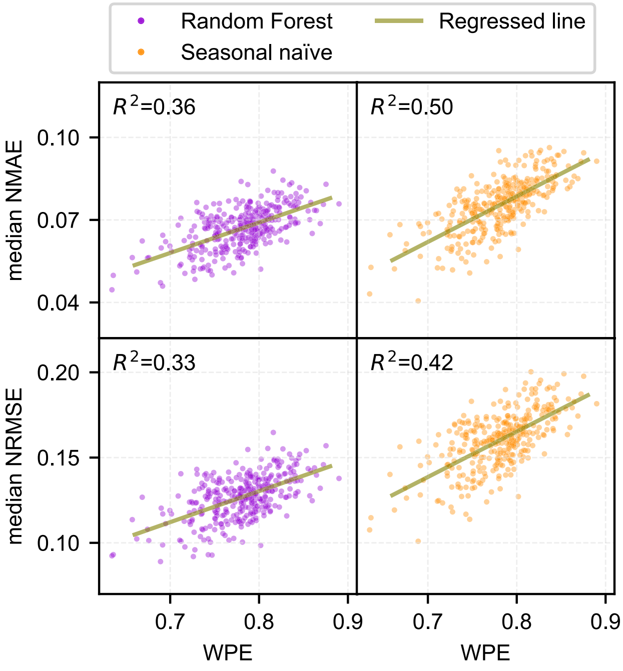

Figure 4. Strong correlation between the WPE and the day-ahead prediction errors of solar PV generation

The scatterplots show the relationship between the WPE of dimension 6 and the 10-min resampling interval, and the median NRMSE and the median NMAE of the day-ahead predictions of PV generation data using random forest and seasonal naive forecasting methods.

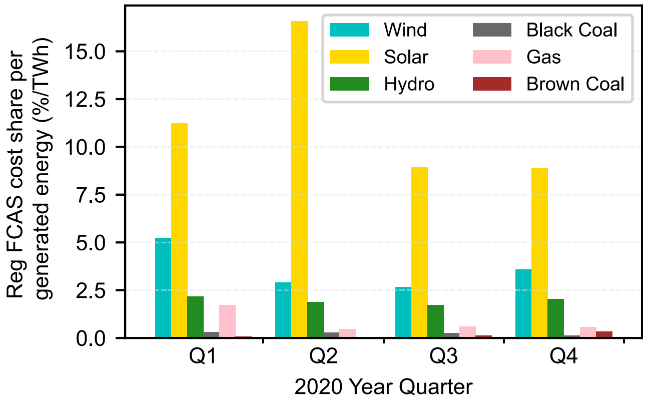

Figure 5. Australian electricity market regulation costs by type of power plant

Each bar illustrates the share of power plants with a particular fuel type in the regulation FCAS costs of the Australian electricity market in each quarter of 2020. The shares of costs in each quarter are normalized by the terawatt-hours of generated energy during that period. The data for the regulation FCAS costs and the generation of each fuel type are collected from AEMO[78] and OpenNEM,[79] respectively.

revenue of about AUS$10,000 per MW installed capacity in 2020.[59] Accordingly, we calculated the revenue of a 51.8 MW solar farm in other locations, assuming a 1% higher annual GTI would lead to 1% more revenue. Also, using the most conservative estimate for the slope of the regressed line between the CPF and the predictability, shown in Figure 7B, it is possible to estimate the reduction in the CPF when the predictability increases. For instance, the figure shows that a 0.1 increase in the renewable generation predictability would reduce CPF by 0.272. Given that the average annual cost of the regulation market was about $85.6 million in the last 5 years,[60] a 0.272 reduction in the CPF leads to roughly $233,000 lower cost of regulation FCAS each year. Finally, the net revenue of the solar farm in different locations is compared in Figure 9. According to the bar plot, location 9 would be the best option for building a 51.8 MW solar farm without considering the predictability. However, if the cost associated with the predictability of PV generation were taken into account, location 5 would be the best option by a significant margin over location 9. Once predictability is considered, the ranking of the choices changes significantly in relation to the highest net revenue, suggesting it has a considerable impact on solar farm investments.

These observations indicate that considering generation predictability as a factor in investment decisions of renewable plants is imperative because profits strongly depend on it. Figure 10 illustrates the changes in the predictability of solar PV generation in various locations in Australia. We can observe that the PV generation predictability varies significantly across different regions and even within each state, indicating that it is highly location dependent. Based on the previous analysis, we can estimate that a 100 MW solar farm could lose roughly $900,000 of its revenue each year because of these differences in PV generation predictability (this would be 9% of its potential $10 million revenue). Without considering predictability, investment studies for building renewable plants will produce suboptimal results. In other words, to find the best locations for future solar PV and wind farm projects, we must take into account the cost implications of the predictability as a decisive factor in addition to the other technical and financial factors currently being used.[50,51] This can be done by measuring the predictability of the potential renewable generation at a location using the existing (or simulated) generation data or relevant surrogate variables, such as GTI for solar PV farms. This is particularly vital, since the predictability measure is not correlated with other factors being considered in such studies. For further discussion on this topic, please refer to Note S1.

The predictability of renewable generation is expected to play an increasingly important role in the future. Renewable energy plants are currently subject to different regulations than conventional ones by electricity market operators under the direction of regulators and policymakers. For example, in Australia, conventional generators are noncompliant if their generation deviates from their instructed dispatch target; renewable plants are not.[61,62] As most conventional power plants are expected to retire in the next few years in Australia, the market rules have been changing to ensure reliable grid operation. Such changes mean that if renewable plants' output is not predictable, they must leave sufficient headroom (i.e., not generate at their maximum availability) to respond to the unpredictable generation changes and meet their forecasts. Otherwise, they must invest in storage systems to ensure that their power plants comply with the rules or face heavy penalties. Both can be costly solutions for investors but can be mitigated by choosing a location with high generation predictability.

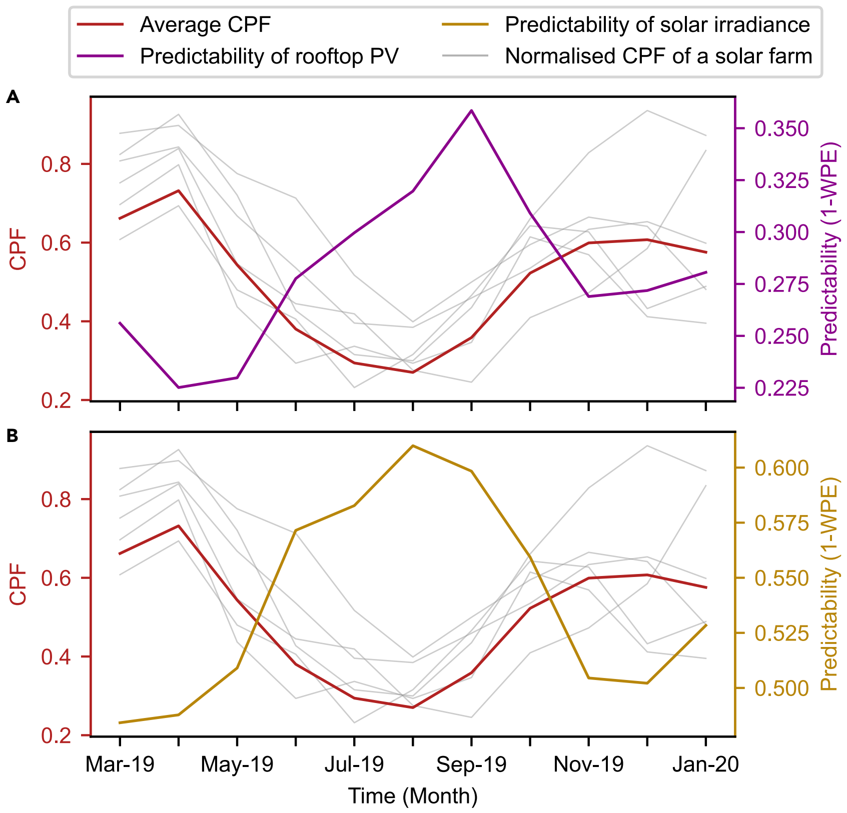

Figure 6. Dependency of solar farms' regulation market costs on the predictability of PV generation

These plots show the relationship over time between the predictability (1 − WPE) of PV generation and the solar farms' causer pays factors (CPFs) in New South Wales (NSW) over the year 2019. In (A), the PV generation data in NSW from our dataset is used to calculate the average predictability over time. However, in (B), the average predictability of PV generation is determined based on the predictability of global tilted irradiance (GTI) sun-tracking data in the exact locations of the six solar farms in NSW. The predictability of PV generation is calculated over 2-month rolling horizons with 1-month shifting forward. To make the CPF data comparable with the predictability values, we calculate the 2-month moving average of the CPF values of solar farms in the same way. The CPF data[57] of solar farms in 2019 is gathered for every solar farm in NSW with a generation capacity above 10 MW, which are commissioned in or before January 2019, namely Griffith, Royalla, Mugga Lane, Manildra, Coleambally, and Moree solar farms. Also, the average CPF of solar farms and the predictability of monthly GTI data are calculated by the weighted average of the six farms based on their maximum generation capacity.

Policy design

The unpredictability of renewable generation, caused by inherently uncertain weather forecasts, has increased the costs of reserve electricity markets.[63,64] For instance, the United Kingdom power grid experiences a £5–10 per MWh increase in the reserve market costs for 1 MW additional wind or solar production due to their prediction errors. As the number of renewable plants increases in the electricity grid, the additional cost of operational reserve requirements per MWh of renewable energy will rise even more.[63] Given that consumers (or taxpayers) pay for these costs on their electricity bills (or government subsidies), governments are responsible for minimizing these costs through well-planned investments and shrewd policy design.

In November 2021, SA became the first gigawatt-scale power grid in the world to reach zero net demand when the combined output of rooftop solar and other small-scale generators exceeded the total customers' load demand.[65] This has been achieved mainly by the federal government's subsidies on rooftop PV panels[66] as well as state government policies, such as solar feed-in payments.[67,68] Despite all the benefits, the high level of distributed PV generation has led to higher variability of power system net demand, which can cause high spot energy prices, voltage swings, or even loss of supply if not adequately managed.[69] One well-known solution is to integrate costly battery storage systems in the grid. Yet a cheaper but effective way to mitigate some of these issues is to invest in renewable energy sources with higher generation predictability.

In this regard, recognizing the differences in the predictability of renewable generation in different areas could change the public sector policies for the better long-term good. For instance, as shown in Figure 11, northern SA with the highest predictability has an average density of rooftop PV systems equal to 41.5%, comparable with the south (40%) with significantly lower predictability. A better policy could have been offering different incentives in different regions based on their predictability, e.g., rooftop PV only in the northern part of the state and PV plus battery in the south.

On a larger scale, considering renewable generation predictability as a factor in the decision-making processes can impact the strategies for dealing with the uncertain nature of renewable generation. For instance, the lower predictability of PV generation in the state of VIC (Figure 10) suggests that increasing rooftop solar PV in that region will increase net demand uncertainty compared with other states, which in turn requires a higher amount of operational reserve requirements in the power grid and, hence, higher cost of energy for consumers. This can shape the policies and regulations to push for alternative renewable generation (e.g., small-scale wind turbines) instead of rooftop PV or subsidies on home battery systems in that region. These are only a few examples to showcase the significance of considering PV generation predictability in various aspects of electricity industry policymaking.

Other applications in power systems

Apart from the application of predictability in private investment and policy design, we identified several potential applications related to other aspects of the energy sector, such as power system daily operation, power grid planning studies, and even automated diagnosis of generation anomalies in a large number of PV systems. For instance, considering the

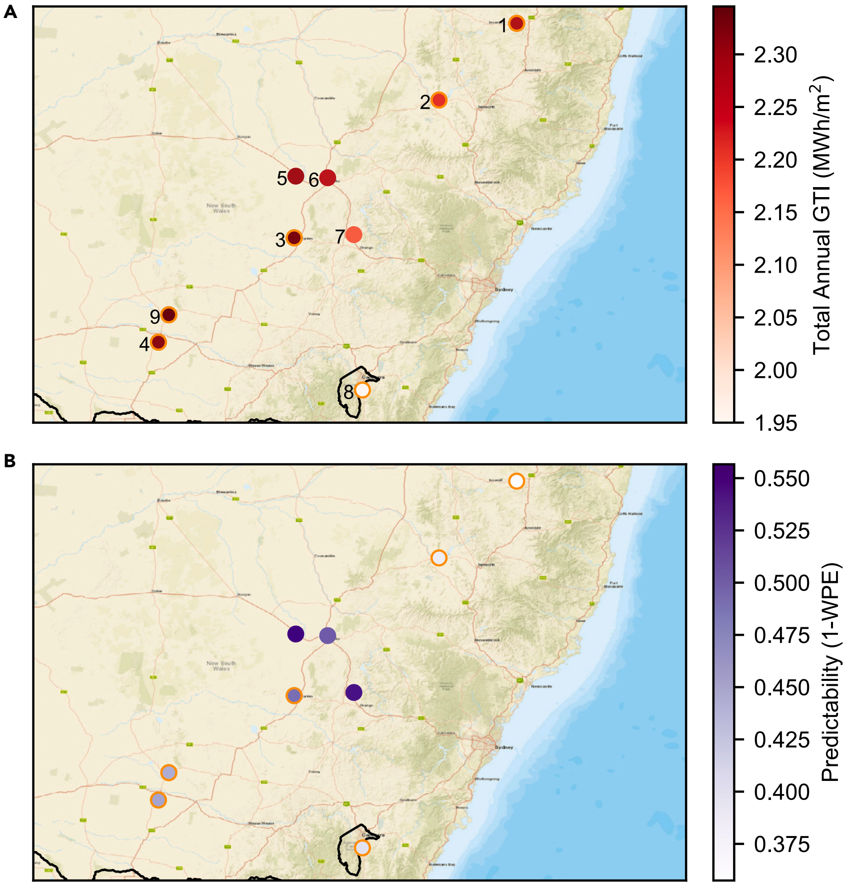

Figure 7. Strong inverse correlation between the regulation market costs and the generation predictability of solar farms

In (A), the scatterplots illustrate the relationship between the average predictability and CPF of 2-month rolling horizons for the rooftop PV generation and the GTI dataset (the same data as in Figure 6). In (B), the scatterplot shows the relationship between the predictability of the solar farms' GTI data and CPF for the six solar farms in NSW in the year 2019. The CPFs are normalized based on the capacity of each solar farm. In all scatterplots, the correlation between the two variables is statistically significant ($p < 0.05$); thus, the R^2 value is shown. Also, in each plot the linear regression equation describing the quantitative relationship between the CPF and the predictability is presented using the ordinary least squares method.

predictability changes over time reveals interesting and helpful seasonality patterns in renewable generation predictability that can advise system planning studies. Figure 12 presents the predictability profiles of the rooftop PV generation in three states of Australia, namely NSW, VIC, and SA, over time. In these charts, each line represents the predictability profile of an Australian household over the year 2019, calculated on the basis of 2-month rolling windows that move forward 1 day at a time. It can be seen that the evolution of predictability over time varies remarkably from one state to another. This observation may have significant policy design implications, e.g., to jointly plan future flexibility resources (such as batteries or pumped hydro storage) and interconnections between states to share flexibility capacity in different seasons, thus lowering the cost of decarbonization for all Australians. For example, the PV generation predictability is the lowest in SA from August to October (Figure 12A) but the highest in NSW during the same period (Figure 12C). In the event of proper interconnection between the two states (in May 2021, the Australian Energy Regulator approved constructing a new interconnector between SA and NSW[70]), the flexibility sources in NSW can be used to manage the higher uncertainties in the SA power grid during that time. The average predictability of renewable generation in each state can also inform power system operators and market participants in determining the time frame for the annual maintenance of their assets, ensuring the availability of enough reserve requirements when renewable resources have lower predictability. Lastly, acknowledging that the maximum capability of prediction methods varies in different states over time can advise power system operators regarding the forecasting accuracy of renewable sources in different areas and, thus, better estimations for required frequency regulation sources in the power systems.

By knowing that solar PV generation is highly correlated with solar insolation, we expect to see similar output from the PV systems located in close proximity. As a result, we should observe similar changes in the predictability of the PV systems in the same region, which can be verified in Figure 12. This further proves that the predictability of the PV generation, measured by the WPE, is an inherent feature of the PV system. From a different perspective, we can use this property to automatically diagnose faults, shading, and other inefficiencies and malfunctions in PV systems within a region using "big data" analysis.[71]

DISCUSSION

The overwhelming attention to improving the forecasting methods of renewable generation in recent years has overshadowed the fact that no method can provide perfect predictions for renewable generation. This leads us to think about how the inherent predictability of renewable generation can be quantified. In this paper, we tried to shed light on this missing piece of the puzzle. By conducting a set of analyses on an actual PV generation dataset, we found a reliable method among various potential metrics to quantify the inherent predictability of renewable generation data. For this purpose, we demonstrated that the WPE with dimension 6 and a 10-min resampling interval is the most suitable predictability measure for our application, illustrating its strong relationship with forecast errors regardless of the forecasting method, prediction horizon, and error metric. Then, using our PV generation dataset and analyzing various

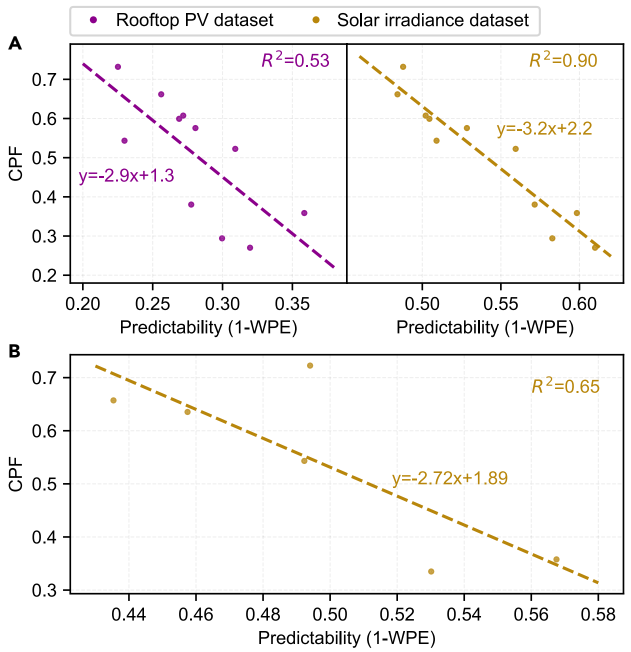

Figure 8. Annual solar irradiance and predictability values of (potential) solar farms in NSW

In (A), the fill color of the circles quantifies the total annual GTI sun-tracking values for the potential solar farm locations in NSW. In (B), the fill colors of the circles quantify the predictability (1 − WPE) of the GTI data at the potential solar farm locations in NSW. The circles with orange borders indicate actual farm locations. Both the annual solar irradiance and predictability are calculated based on the 5-min GTI time series from August 2021 to August 2022. The GTI data were obtained from SolCast.[58].

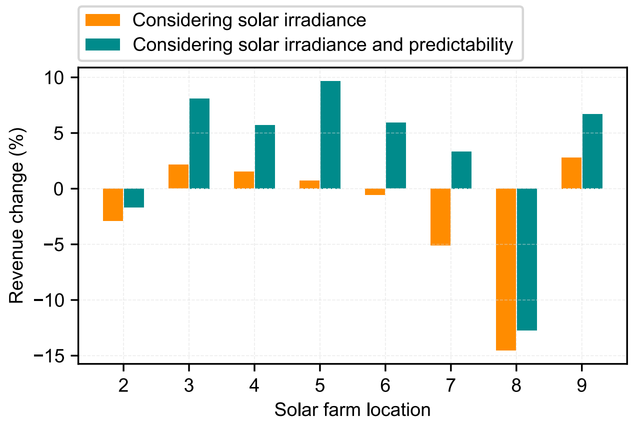

Figure 9. Impact of considering the predictability in choosing the best location for building a solar farm

The bar plot shows the projected revenue changes of a 51.8 MW solar farm when installed in different locations, shown in Figure 8, with respect to location 1. In the first scenario, the revenue only depends on the annual solar irradiance. In the second scenario, the costs associated with the regulation market costs are also taken into account based on the changes in the predictability; thus, the CPF.

real-world examples, we provided evidence that this measure can offer valuable additional information to decision-makers in the energy industry. Revealing the significant impact of PV generation predictability on the profit of solar farms, we demonstrated that considering the predictability in renewable plant investments can lead to better decisions with higher profits. Also, by comparing the rooftop PV density and predictability data in SA, we showed that policymakers can benefit from considering renewable generation predictability in policy design. Lastly, we demonstrated how predictability can be applied beyond what we discussed here, analyzing PV generation predictability in different states of Australia to show an example of such applications.

Electricity generation and consumption are undergoing significant changes, for example the ever-increasing adoption of electric vehicles and rooftop PV systems combined with the installation of utility-scale renewable power plants, making electricity supply and demand more unpredictable. Consequently, estimating the predictability of generation and demand is becoming more critical than ever. While forecasting methods are essential, they will never be perfect. Hence, attention must be paid to the other half of the problem: the limited inherent predictability of intermittent generation sources. Measuring this property of renewable generation data can offer numerous direct and indirect insights to policymakers, investors, power system planners and operators, and third-party service providers in the electricity industry for better decision-making.

While the analysis in this paper is based on PV generation data, future studies on wind energy generation might gain similar insights. Also, even though the predictability measure chosen for this paper works well for measuring the short-term predictability of renewable energy, it cannot be used to measure long-term predictability (months and years ahead). A metric for estimating long-term predictability could have many applications in the electricity industry, e.g., quantifying the long-term risks associated with renewable energy investments. Future research can benefit from addressing these limitations.

EXPERIMENTAL PROCEDURES

Resource availability

Lead contact

Further information and requests for resources should be directed to and will be fulfilled by the lead contact, Sahand Karimi-Arpanahi (sahand.karimi-arpanahi@adelaide.edu.au).

Materials availability

There are no physical materials associated with this study.

Data and code availability

All the Python codes used for the mentioned analyses in this study are available on Zenodo (https://doi.org/10.5281/zenodo.7538884)[72] and a GitHub repository (https://github.com/sahand-karimi/Measuring_Predictability_Renewable_Energy). The original rooftop PV generation data from Solar Analytics, used in this study, cannot be shared because of a nondisclosure agreement with the company. However, we have added a synthetic dataset with a similar structure to our rooftop PV generation to the repository. This dataset is synthesized by interpolating hourly solar irradiance data to 5-min resolution in different locations of Australia in 2015.[73] While this synthetic dataset does not meet the criteria for the application described in our study, it guides the users to prepare their own dataset in the correct structure that can be used by our code. Also, it can be used as an example to study the code. Please note that a renewable generation dataset should satisfy the following three conditions for the applications described in this paper: (1) it should have at least a temporal resolution of 10 min (it can differ depending on the electricity

CellPress
OPEN ACCESS

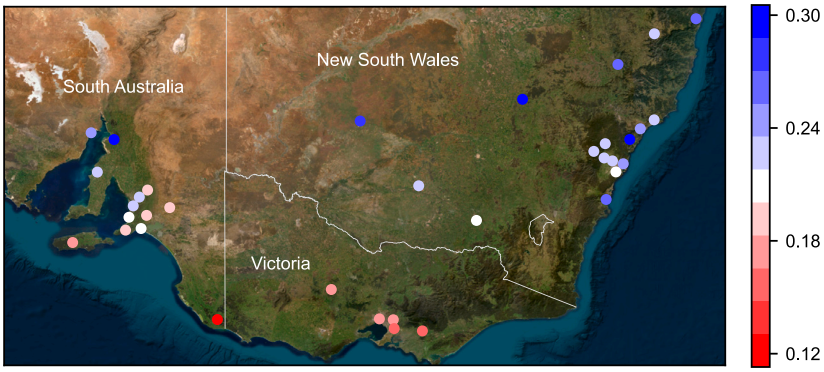

Figure 10. Impact of location on the predictability of solar PV generation

The predictability values are shown for the 1-year PV generation of houses in different regions across Australia. Each region consists of postcodes within 25 km of each other. The circles on the maps are colored based on the average predictability (1 − WPE) of PV systems in that region. The resampling interval of the PV generation time series is 10 min, and the embedding dimension in the WPE calculation is set to 6.

market and compliance rules); (2) the measured or estimated data should not be systematically affected by the external factors, such as generation curtailment, export limits, and so forth; and (3) the length of data should be at least 6 months, and the dataset should cover a wide geographical area such as a country.

The solar irradiance data (GTI) from SolCast, used in this study, cannot be shared publicly, but university students and researchers can freely access the data on SolCast.com to reproduce our results.[58] To do so, one should create a "Student or public researcher" account and then submit a "Time series request" with the following details. Enter all the locations of the (potential) solar farms as in our study (the exact latitude and longitude of each location are available in the public repository). Set the "Data period" as mentioned in the relevant analysis, "Time granularity" to 5 min, and "File format" to SolCast. Select "GTI horizontal single-axis tracker" as one of the parameters in the request. Finally, download the GTI data and use it as input for the relevant analyses. In the case of an issue in accessing these data, please reach out to the lead contact.

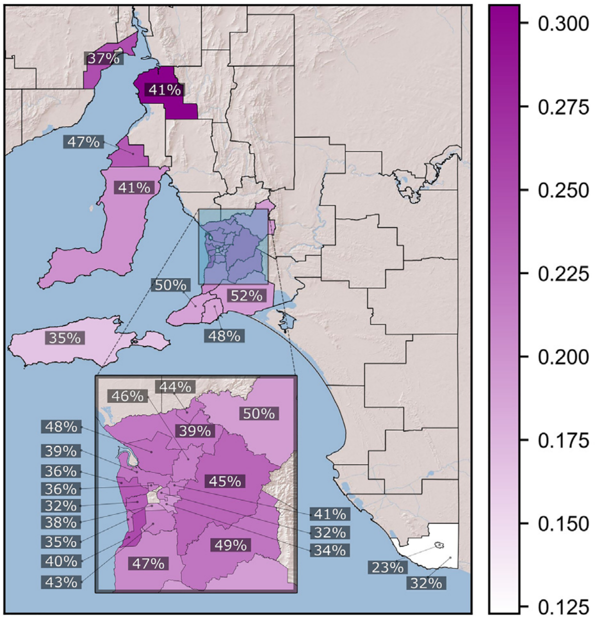

Figure 11. Predictability and density of rooftop PV generation in SA

A comparison between the PV generation predictability at SA's local government areas (LGAs) with available data and the density of the dwellings with rooftop PV in the LGAs. The data for rooftop density is obtained from the Australian PV Institute.[80] Also, the resampling interval of the PV generation time series is 10 min, and the embedding dimension of WPE is 6.

Permutation entropy

Aiming to define a predictability measure, which is "easily calculated for any types of time series, be it regular, chaotic, noisy, or reality-based," Bandt and Pompe introduced PE in their seminal work.[37] As a model-free complexity metric, PE was shown to behave similarly to Lyapunov exponents and Kolmogorov-Sinai entropy (i.e., the complexity measures of dynamical systems).[37,43,74] PE can readily be calculated by determining the ordinal pattern of the vectors in a time series and extracting the probability distribution of the ordinal patterns. The core idea in PE is that the patterns inside a time series do not have a similar probability of occurrence. For instance, if a couple of specific patterns occur most often in a time series, PE will be a small number close to zero, meaning that the time series is predictable because of the repetitive patterns. Conversely, if all patterns have an almost equal probability of occurrence, PE will be close to 1, showing that the time series is difficult to predict.

To calculate the PE of time series $\{x_t\}_{t=1,\ldots,N}$ of length N for embedding dimension d, we first divide the time series into $N-(d-1)$ embedding vectors (i.e., sequences of values) of length d, which are $X_t^d=(x_t,x_{t+1},\ldots,x_{t+(d-1)})$ for $t=1,\ldots,N-(d-1)$. We then assign each vector to a single permutation, π_i, in the set of possible permutations, Π, which includes all possible unique orderings of d real numbers. Therefore, there are $d!$ unique permutations in Π. In other words, we associate each sequence of values, X_t^d, to one permutation, π_i, based on the sequence's ordinal pattern, $\pi_i \sim \phi(X_t^d)$. For example, if X_t^d is {4,3,7}, then $\phi(X_t^d)$, the ordinal pattern of this sequence, is 2-1-3.

For each $\pi_i \in \Pi$, the relative frequency of permutation π_i occurring in time series $\{x_t\}$ is

$$P(\pi_i)=\frac{\left|\{t,|,t<N-d,\phi(X_t^d)=\pi_i\}\right|}{N-(d-1)}, \quad \text{(Equation 1)}$$

where $|.|$ shows cardinality, $\phi(X_t^d)$ is the ordinal pattern of X_t^d, and $P(\pi_i)$ is the occurrence probability of vectors that has the same ordinal pattern as permutation π_i.

Using the above definitions, PE for $d \geq 2$ is defined as

$$PE(d)=-\sum_{\pi_i \in \Pi} P(\pi_i)log_2(P(\pi_i)). \quad \text{(Equation 2)}$$

As $0<PE(d)<log_2(d!)$, the PE value is commonly normalized by dividing it by $log_2(d!)$, so the normalized PE values are between 0 and 1. This way, the values of PE in different dimensions are comparable with each other. In this paper, the normalized PE is referred to as the "PE."

It is also worth noting that the Bandt and Pompe[37] recommended that, for practical purposes, the embedding dimension should be a number between 3 and 7 ($d \in \{3,4,5,6,7\}$). Additionally, to allow all possible patterns in the time series to appear in the analysis, we should select the dimension such that $d! \ll N$. This enables accurate estimation of the relative frequency of permutations for a finite time series. Note that to determine the exact values of the frequencies, we must have $N \rightarrow +\infty$.[75] Lastly, because PE does not consider the possibility of equal values in a vector, tie-breaking methods should be implemented in those situations, particularly in discrete-valued time series. However, such circumstances can infrequently happen if the data are recorded in high resolution, as in our dataset.

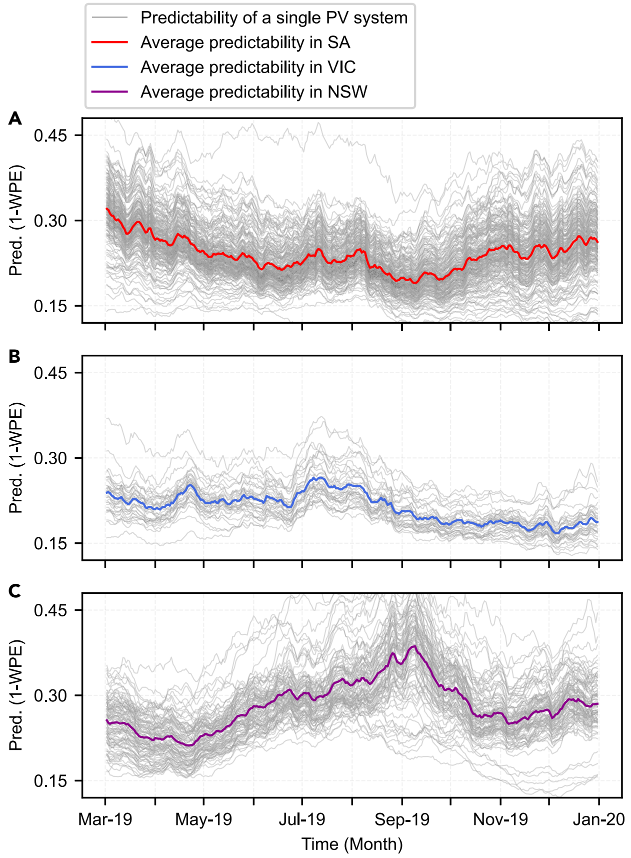

Figure 12. Different patterns of changes in PV generation predictability over time in different states of Australia

The predictability values (1 − WPE) of a 2-month rolling horizon across a year for the PV systems' generation profiles in the states of (A) SA, (B) VIC, and (C) NSW. In this analysis, the resampling interval of the PV generation time series is 10 min, and the embedding dimension of the WPE is 6.

Weighted permutation entropy

A major drawback in PE for measuring the predictability of time series is that it reflects no information other than the order structure of the vectors in a time series. This is particularly significant when the changes in the amplitude of a time series contain important information, such as in a PV generation time series. In addition, PE is highly sensitive to measurement when the values of the observations in the time series are close to each other. To deal with these issues, an improved version of PE was proposed by Fadlallah et al.,[43] whereby they assigned a weight to each vector in the time series, calling the new measure WPE. It is less sensitive to noises and considers the amplitude information of the observations in the calculations. This is because the weights of the vectors are quantified according to the variance of the observations in each vector.

To calculate the WPE of a time series, we first determine the weight of each vector as follows:

$$w_t = \frac{1}{d}\sum_{s=1}^{d}\left(x_{t+(s-1)} - \overline{X}_t^d\right)^2, \quad \text{(Equation 3)}$$

where $\overline{X}_t^d$ is the arithmetic mean of the values in the corresponding vector.

For each $\pi_i \in \Pi$, the weighted probability of permutation π_i occurring in time series $\{x_t\}$ is

$$P_w(\pi_i) = \frac{\sum_{t=1}^{N-(d-1)}\left(w_t.\psi\left(\phi,\left(X_t^d\right),\pi_i\right)\right)}{\sum_{t=1}^{N-(d-1)} w_t}, \quad \text{(Equation 4)}$$

where $\psi(a,b)$ is 1 when $a = b$, and 0 otherwise.

Using the above definitions, WPE for $d \geq 2$ is defined

$$WPE(d) = -\sum_{\pi_i \in \Pi} P_w(\pi_i) log_2(P_w(\pi_i)). \quad \text{(Equation 5)}$$

Similar to PE, WPE values are normalized by $log_2(d!)$. In this paper, the normalized WPE is referred to as the "WPE."

Sample entropy

Pincus adapted the concept of entropy for real-world applications by proposing approximate entropy (ApE) to measure the complexity of time series. He argued that the variations in the proposed measure are closely related to the changes in Lyapunov exponents and Kolmogorov-Sinai entropy, thereby demonstrating the ability of ApE to indicate the complexity of system dynamics by using imperfect and finite data.[35]

To determine the ApE for time series $\{x_t\}_{t=1,\ldots,N}$, we should first form a sequence of vectors $\{u_t^m\}_{t=1,\ldots,N-m+1}$ with length m, where $u_t^m = [x_t, x_{t+1}, \ldots, x_{t+m-1}]$ and m is a positive integer (for the sake of consistency with PE and WPE definitions, we call m "dimension"). Second, we define function $d(u_t^m, u_\tau^m)$ as the maximum of the absolute values of the component-wise differences between the two vectors. Next, we count "similar" vectors with dimension m in the time series by

$$C_t^m(r) = \frac{1}{N-m+1}\left|\left\{\tau \middle| d\left(u_t^m, u_\tau^m\right) < r\right\}\right|, \quad \text{(Equation 6)}$$

where $|.|$ shows cardinality, and r specifies the tolerance for two vectors to be considered similar (we call r "match criterion"). $C_t^m(r)$ is actually the probability that vector u_τ^m is within r of u_t^m. Using this function, we next define

$$\Phi^m(r) = \frac{1}{N-m+1}\sum_{t=1}^{N-m+1} ln\, C_t^m(r). \quad \text{(Equation 7)}$$

Considering the mentioned definitions, the ApE of time series $\{x_t\}_{t=1,\ldots,N}$ is defined as

$$ApE(m,r) = \lim_{N\to+\infty}\left(\Phi^m(r) - \Phi^{m+1}(r)\right). \quad \text{(Equation 8)}$$

Owing to some issues in the practical implementation of ApE, Richman et al.[36] proposed an improvement to the ApE, defining it as sample entropy. An important shortcoming of the ApE is its bias in the calculation of $C_t^m(r)$, since the self-matches of the template vector, u_t^m, are included. This would particularly be an issue for relatively short time series.[39] To address this issue in calculating $C_t^m(r)$, they did not consider self-matches. In addition, they considered only the first $N-m$ vectors of dimension m (instead of $N-m+1$ in ApE) to ensure that, for $1 \leq t \leq N-m$, both u_t^m and u_t^{m+1} are defined. Therefore, to calculate the SaE, we first define

$$B_t^m(r) = \frac{1}{N-m-1}\left|\left\{\tau \middle| d\left(u_t^m, u_\tau^m\right) < r, t \neq \tau\right\}\right|, \quad \text{(Equation 9)}$$

$$A_t^m(r) = \frac{1}{N-m-1}\left|\left\{\tau \middle| d\left(u_t^{m+1}, u_\tau^{m+1}\right) < r, t \neq \tau\right\}\right|. \quad \text{(Equation 10)}$$

Thereafter, we define

$$B^m(r) = \frac{1}{N-m}\sum_{i=1}^{N-m} B_t^m(r), \quad \text{(Equation 11)}$$

$$A^m(r) = \frac{1}{N-m}\sum_{i=1}^{N-m} A_t^m(r). \quad \text{(Equation 12)}$$

Considering the above definitions, the SaE of time series $\{x_t\}_{t=1,\ldots,N}$ is defined as

$$SaE(m,r) = \lim_{T\to+\infty} -\ln\left(\frac{A^m(r)}{B^m(r)}\right), \quad \text{(Equation 13)}$$

which can be estimated by statistic $SaE(m,r) = -\ln\left(\frac{A_m(r)}{B^m(r)}\right)$.

The SaE is more robust against data length and displays relative consistency under different circumstances than the ApE. Also, it can be calculated faster than ApE because of its higher computational efficiency.[36]

Dispersion entropy

Tackling some limitations of SaE and PE, Rostaghi and Azami introduced DE in 2016 as a new "irregularity indicator." Since the probability of different states is similar in a system with maximum entropy or irregularity, it is impossible to predict the system states. Conversely, the system has a minimum irregularity (or entropy) if there is only one state with the probability of one that can happen.[45]

Accordingly, to calculate the DE of time series $\{x_t\}_{t=1,\ldots,N}$ of length N for embedding dimension d and classes c, we first map each data point x_t to one of the classes, labeled from 1 to c. While this mapping can be done through different linear or nonlinear methods, the typical way[45] is to employ the normal cumulative distribution function to map $\{x_t\}$ to $\{y_t\}_{t=1,\ldots,N}$, where y_t is between 0 and 1. We can then use a linear algorithm to assign y_t to an integer from 1 to c, creating $\{z_t\}_{t=1,\ldots,N}$. Then, similar to PE, we first divide the time series into $N-(d-1)$ embedding vectors (i.e., sequences of values) of length d, which are $Z_t^d = (z_t, z_{t+1}, \ldots, z_{t+(d-1)})$ for $t = 1,\ldots,N-(d-1)$. We then assign each vector to a single dispersion pattern among all possible patterns. The number of possible dispersion patterns is equal to c^d, since the length of each embedding vector is d, and each data point in the vector can be an integer from 1 to c. Thereafter, similar to PE (Equations 1 and 2), the relative frequency of each dispersion pattern is calculated, based on which the value of DE is determined. Lastly, as this value would be between 0 and $log_2(c^d)$, it is normalized by dividing it by $log_2(c^d)$. This paper refers to the normalized DE as the "DE."

Spectral entropy

The power spectral density (PSD) (or simply power spectrum) of a signal, widely used in signal-processing literature, describes the distribution of the signal's power content based on the frequency components composing the signal.

To calculate the SpE, we first utilize a periodogram to estimate the PSD of the time series. The PSD is then normalized by the total power of the time series. Thus, there would be $\sum_{f=0}^{f_n} S_f = 1$, where S_f is the normalized power spectrum, and f_n is a reasonably high frequency.[76]

Given the normalized PSD of the time series, its SpE can be defined as

$$SpE = -\sum_{f=0}^{f_n} S_f \log_2(S_f). \quad \text{(Equation 14)}$$

Predictability

As discussed earlier in the paper, predictability is an inherent property of dynamical systems.[23] To measure the predictability of time series $\{x_t\}_{t=1,\ldots,N}$ using its entropy, we define it as

$$Pred. = max(H) - H(\{x_t\}), \quad \text{(Equation 15)}$$

where H denotes the entropy value and $max(H)$ is the maximum value that it can have. Thus, if the entropy is normalized, $max(H) = 1$.

Time series resampling

Time series resampling is an essential technique that allows us to flexibly find the best time resolution for our analysis. It can be used for different purposes, e.g., handling large datasets, removing the impact of sporadic measurement errors, or getting desirable results for specific purposes.

To perform resampling, we created each data point of the resampled time series based on the average of the required number of data points in the original time series. For instance, to create a 10-min resampled time series from our original 5-min data, we created each data point of the resampled time series based on the average of two data points in the original series.

Normalized root-mean-square error

Assume A_t,F_t, and e_t respectively denote actual, forecast, and error values for $t = 1,\ldots,N$. Then, the NRMSE will be calculated as follows:

$$e_t = A_t - F_t : \forall t \in \{1,\ldots,N\}, \quad \text{(Equation 16)}$$

$$RMSE(RootMeanSquareError) = \sqrt{mean(e_t^2)}, \quad \text{(Equation 17)}$$

$$NRMSE = \frac{RMSE}{max(A_t) - min(A_t)}. \quad \text{(Equation 18)}$$

Please note that in the minutes-ahead predictions, NRMSE is normalized by the 99th percentile to avoid the impact of potential measurement errors on the normalization.

Normalized mean absolute error

Assume A_t,F_t, and e_t respectively denote actual, forecast, and error values for $t = 1,\ldots,N$. Then, the NMAE will be calculated as follows:

$$e_t = A_t - F_t : \forall t \in \{1,\ldots,N\}, \quad \text{(Equation 19)}$$

$$MAE(MeanAbsoluteError) = mean(|e_t|), \quad \text{(Equation 20)}$$

$$NMAE = \frac{MAE}{max(A_t) - min(A_t)}, \quad \text{(Equation 21)}$$

where $|.|$ shows the absolute function.

Please note that in the minutes-ahead predictions, NMAE is normalized by the 99th percentile to avoid the impact of potential measurement errors on the normalization.

ARIMA prediction method

We used ARIMA as one of the standard minutes-ahead prediction methods to determine the prediction errors of time series in the datasets. ARIMA works on the basis of autocorrelations in the time series to model temporal structures. We can then use the fitted model to predict future values in a time series. The ARIMA model consists of three main components, based on which a model is fitted on a time series. The autoregression (AR) part of ARIMA aims to describe a particular time series data point based on the linear regression of past observations. The moving average (MA) part uses past prediction errors in the time series in a regression-like model to predict future values. Lastly, if the time series is not stationary, the integrated (I) part of ARIMA calculates the differences between consecutive observations of the time series to create a stationary time series, using which the model would be trained. Interested readers are referred to the book by Hyndman and Athanasopoulos[27] for additional information regarding the ARIMA models.

Naive prediction method

The naive prediction method was used as the second minutes-ahead representative prediction method in our analysis. As a result of this method, forecast values are set to previous period observations, i.e.,

$$F_{T+t|T} = A_T, \quad \text{(Equation 22)}$$

where A_t and F_t denote the actual and predicted values, respectively, and $F_{T+t|T}$ is the predicted value at time $T+t$ when A_T is the last historical data value.

Random forest prediction method

We used random forest regression as one of the common day-ahead prediction methods to determine the prediction errors of time series in the datasets. Random forest regression is a supervised learning algorithm that uses an ensemble learning method for regression. This method combines predictions from multiple machine-learning algorithms to make more accurate predictions than those of a single model using random forecast as a regressor.[77]

Seasonal naive prediction method

This article used the seasonal naive prediction method as the second day-ahead representative prediction method. This method sets predicted values to the observed values for the previous seasonal period, where the seasonal period is 1 day for the PV generation time series. Accordingly, the predicted values are calculated by

$$F_{T+t|T} = A_{T+t-m(k+1)}, \qquad \text{(Equation 23)}$$

where A_t and F_t denote the actual and predicted values, respectively; $F_{T+t|T}$ is the predicted value at time $T+t$ when A_T is the last historical data value; m is the seasonal period; and k is the integer part of $(t-1)/m$.

SUPPLEMENTAL INFORMATION

Supplemental information can be found online at https://doi.org/10.1016/j.patter.2023.100708.

ACKNOWLEDGMENTS

This research project is supported by the School of Electrical and Mechanical Engineering at The University of Adelaide and a supplementary scholarship provided by the Commonwealth Scientific and Industrial Research Organisation (CSIRO) of Australia, grant number 55129767. This work was supported with supercomputing resources provided by the Phoenix HPC service at the University of Adelaide. We also thank SolCast for providing us with the historical solar irradiance data used in this study.

AUTHOR CONTRIBUTIONS

S.K.-A. and S.A.P. conceived and designed this research project and gathered the data. S.K.-A. curated the data, developed the codes, conducted the analyses, produced the figures, and wrote the first draft of the manuscript. S.K.-A., S.A.P., and N.M. discussed the technical details, analyzed the results, and reviewed and edited the final manuscript. S.A.P. and N.M. supervised this research and acquired funding.

DECLARATION OF INTERESTS

The authors declare no competing interests.

INCLUSION AND DIVERSITY

We support inclusive, diverse, and equitable conduct of research.

Received: November 5, 2022
Revised: January 15, 2023
Accepted: February 21, 2023
Published: March 24, 2023

REFERENCES

1. World energy outlook 2022. (2022) (International Energy Agency), IEA. https://www.iea.org/reports/world-energy-outlook-2022.
2. End of coal in sight as UK secures ambitious commitments at COP26 summit. (2021). https://www.gov.uk/government/news/end-of-coal-in-sight-as-uk-secures-ambitious-commitments-at-cop26-summit.
3. Delarue, E., and Morris, J. Renewables intermittency: operational limits and implications for long-term energy system models. MIT Joint Program on the Science and Policy of Global Change (2015). URL: https://globalchange.mit.edu/sites/default/files/MITJPSPGC_Rpt277.pdf.
4. Managing Large-Scale Penetration of Intermittent Renewables. (2012) (MIT Energy Initiative). https://energy.mit.edu/wp-content/uploads/2012/03/MITEI-RP-2011-001.pdf.
5. South Australia's Energy Solution. (2020) (Government of South Australia). https://www.energymining.sa.gov.au/__data/assets/pdf_file/0009/364266/200615_Energy_Solution_Action_Plan_final_spreads_rs.pdf.
6. 2021 Integrated System Planning (ISP) Methodology. (2021) (Australian Energy Market Operator (AEMO)). https://aemo.com.au/-/media/files/major-publications/isp/2021/2021-isp-methodology.pdf.
7. Leading Western Australia's Brighter Energy Future. (2021) (Government of Western Australia). https://www.wa.gov.au/system/files/2021-07/Energy-Transformation-Strategy-Stage2-July2021.pdf.
8. South Australian generation forecasts. (2021) (Australian Energy Market Operator (AEMO)). https://aemo.com.au/-/media/files/electricity/nem/planning_and_forecasting/sa_advisory/2021/2021-south-australian-generation-forecasts.pdf.
9. Renewable energy resource maps. (2022). https://www.renewablessa.sa.gov.au/investment-portal/renewable-energy-resource-maps.
10. State of the Energy Market 2021 – Chapter 2. (2021) (Australian Energy Regulator). https://www.aer.gov.au/publications/state-of-the-energy-market-reports/state-of-the-energy-market-2021.
11. Okumus, I., and Dinler, A. (2016). Current status of wind energy forecasting and a hybrid method for hourly predictions. Energy Convers. Manag. *123*, 362–371. https://doi.org/10.1016/j.enconman.2016.06.053. https://www.sciencedirect.com/science/article/pii/S0196890416305428.
12. Wang, H., Lei, Z., Zhang, X., Zhou, B., and Peng, J. (2019). A review of deep learning for renewable energy forecasting. Energy Convers. Manag. *198*, 111799. https://doi.org/10.1016/j.enconman.2019.111799. https://www.sciencedirect.com/science/article/pii/S0196890419307812.
13. Ahmed, R., Sreeram, V., Mishra, Y., and Arif, M. (2020). A review and evaluation of the state-of-the-art in PV solar power forecasting: techniques and optimization. Renew. Sustain. Energy Rev. *124*, 109792.
14. Wind power prediction project list. (2021). https://www.ieawindforecasting.dk/other-projects.
15. Half of Australia's solar and wind farms to trial providing their own short-term generation forecasts. (2019). https://www.pv-magazine-australia.com/2019/03/18/half-of-australias-solar-and-wind-farms-to-trial-providing-their-own-short-term-generation-forecasts/.
16. Wind and solar power production forecasts. (2022). https://conwx.com/power-forecasts/wind-and-solar-power-production-forecasts/.
17. Wind power forecasting. (2022). https://enfor.dk/services/windfor/.
18. Short-term wind, solar, and power demand forecasting. (2022). https://www.dnv.com/power-renewables/services/forecaster/.
19. Girard, R., Laquaine, K., and Kariniotakis, G. (2013). Assessment of wind power predictability as a decision factor in the investment phase of wind farms. Appl. Energy *101*, 609–617.
20. Sasaki, W. (2017). Predictability of global offshore wind and wave power. Int. J. Mar. Energy *17*, 98–109.
21. Shahriari, M., Cervone, G., Clemente-Harding, L., and Delle Monache, L. (2020). Using the analog ensemble method as a proxy measurement for wind power predictability. Renew. Energy *146*, 789–801.
22. Makhoul, J. (1975). Linear prediction: a tutorial review. Proc. IEEE *63*, 561–580. https://doi.org/10.1109/PROC.1975.9792.
23. Kumar, A., and Chen, M. (2015). Inherent predictability, requirements on the ensemble size, and complementarity. Mon. Weather Rev. *143*, 3192–3203.
24. Garland, J., James, R., and Bradley, E. (2014). Model-free quantification of time-series predictability. Phys. Rev. E - Stat. Nonlinear Soft Matter Phys. *90*, 052910.
25. Das, U.K., Tey, K.S., Seyedmahmoudian, M., Mekhilef, S., Idris, M.Y.I., Van Deventer, W., Horan, B., and Stojcevski, A. (2018). Forecasting of photovoltaic power generation and model optimization: a review. Renew. Sustain. Energy Rev. *81*, 912–928.
26. Kani, S.A.P., Wild, P., and Saha, T.K. (2020). Improving predictability of renewable generation through optimal battery sizing. IEEE Trans. Sustain. Energy *11*, 37–47.
27. Hyndman, R.J., and Athanasopoulos, G. (2018). Forecasting: Principles and Practice (OTexts).

CellPress
OPEN ACCESS

28. Huang, Y., and Fu, Z. (2019). Enhanced time series predictability with well-defined structures. Theor. Appl. Climatol. *138*, 373–385.
29. Pennekamp, F., Iles, A.C., Garland, J., Brennan, G., Brose, U., Gaedke, U., Jacob, U., Kratina, P., Matthews, B., Munch, S., et al. (2019). The intrinsic predictability of ecological time series and its potential to guide forecasting. Ecol. Monogr. *89*, e01359.
30. Scarpino, S.V., and Petri, G. (2019). On the predictability of infectious disease outbreaks. Nat. Commun. *10*, 1–8.
31. Auer, B.R. (2016). On time-varying predictability of emerging stock market returns. Emerg. Mark. Rev. *27*, 1–13.
32. Crovella, M.E., and Bestavros, A. (1997). Self-similarity in world wide web traffic: evidence and possible causes. IEEE/ACM Trans. Netw. *5*, 835–846.
33. Wu, C., Tang, W., Poolla, K., and Rajagopal, R. (2016). Predictability, constancy and contingency in electric load profiles. In 2016 IEEE International Conference on Smart Grid Communications (SmartGridComm) (IEEE), pp. 662–667.
34. Yang, M., Wang, K., Cui, Y., Feng, F., Su, X., and Ma, C. (2021). Investigating the predictability of photovoltaic power using approximate entropy. Front. Energy Res. *9*, 210.
35. Pincus, S.M. (1991). Approximate entropy as a measure of system complexity. Proc. Natl. Acad. Sci. USA *88*, 2297–2301.
36. Richman, J.S., and Moorman, J.R. (2000). Physiological time-series analysis using approximate entropy and sample entropy. Am. J. Physiol. Heart Circ. Physiol. *278*, H2039–H2049.
37. Bandt, C., and Pompe, B. (2002). Permutation entropy: a natural complexity measure for time series. Phys. Rev. Lett. *88*, 174102.
38. Franzke, C.L.E., Graves, T., Watkins, N.W., Gramacy, R.B., and Hughes, C. (2012). Robustness of estimators of long-range dependence and self-similarity under non-gaussianity. Philos. Trans. A Math. Phys. Eng. Sci. *370*, 1250–1267.
39. Riihijarvi, J., Wellens, M., and Mahonen, P. (2009). Measuring complexity and predictability in networks with multiscale entropy analysis. In IEEE INFOCOM 2009 (IEEE), pp. 1107–1115.
40. Shannon, C.E. (1948). A mathematical theory of communication. Bell System Technical Journal *27*, 379–423.
41. Crutchfield, J.P. (2012). Between order and chaos. Nat. Phys. *8*, 17–24.
42. 2019 Australian voltage and PV generation as part of ARENA grant ARP128. (2019). http://www.solaranalytics.com/data.
43. Fadlallah, B., Chen, B., Keil, A., and Príncipe, J. (2013). Weighted-permutation entropy: a complexity measure for time series incorporating amplitude information. Phys. Rev. E - Stat. Nonlinear Soft Matter Phys. *87*, 022911.
44. Riedl, M., Müller, A., and Wessel, N. (2013). Practical considerations of permutation entropy. Eur. Phys. J. Spec. Top. *222*, 249–262.
45. Rostaghi, M., and Azami, H. (2016). Dispersion entropy: a measure for time-series analysis. IEEE Signal Process. Lett. *23*, 610–614.
46. Colak, H.E., Memisoglu, T., and Gercek, Y. (2020). Optimal site selection for solar photovoltaic (PV) power plants using GIS and AHP: a case study of Malatya Province, Turkey. Renew. Energy *149*, 565–576.
47. Ruiz, H., Sunarso, A., Ibrahim-Bathis, K., Murti, S., and Budiarto, I. (2020). GIS-AHP multi criteria decision analysis for the optimal location of solar energy plants at Indonesia. Energy Rep. *6*, 3249–3263.
48. Villacreses, G., Gaona, G., Martínez-Gómez, J., and Jijón, D.J. (2017). Wind farms suitability location using geographical information system (GIS), based on multi-criteria decision making (MCDM) methods: the case of continental Ecuador. Renew. Energy *109*, 275–286.
49. Rediske, G., Burin, H., Rigo, P., Rosa, C., Michels, L., and Siluk, J. (2021). Wind power plant site selection: a systematic review. Renew. Sustain. Energy Rev. *148*, 111293.
50. Utility-scale Solar Photovoltaic Power Plants: A Project Developer's Guide. (2015) (International Finance Corporation). https://www.ifc.org/wps/wcm/connect/a1b3dbd3-983e-4ee3-a67b-cdc29ef900cb/IFC+Solar+Report_Web+_08+05.pdf.
51. Mehos, M., Price, H., Cable, R., Kearney, D., Kelly, B., Kolb, G., and Morse, F. (2020). Concentrating solar power best practices study. National Renewable Energy Lab.(NREL).
52. Treatment of Loss Factors in the NEM. (2016) (Australian Energy Market Operator (AEMO)). https://aemo.com.au/-/media/files/electricity/nem/security_and_reliability/loss_factors_and_regional_boundaries/2016/treatment_of_loss_factors_in_the_nem.pdf.
53. Settlements Guide to Ancillary Services Payment and Recovery. (2020) (Australian Energy Market Operator (AEMO)). https://aemo.com.au/-/media/files/electricity/nem/data/ancillary_services/2020/settlements-guide-to-ancillary-services-payment-and-recovery.pdf.
54. Gannon, M. (2021). Ranking solar farm FCAS costs in the NEM in 2020. https://wattclarity.com.au/articles/2021/04/ranking-solar-farm-fcas-costs-in-the-nem-in-2020/.
55. Gannon, M. (2021). Ranking wind farm performance and FCAS costs in the NEM in 2020. https://wattclarity.com.au/articles/2021/05/gsd2020-ranking-wind-farm-performance-and-fcas-costs-in-the-nem-in-2020/.
56. Regulation FCAS Contribution Factor Procedure. (2018) (Australian Energy Market Operator (AEMO)). https://aemo.com.au/-/media/files/electricity/nem/security_and_reliability/ancillary_services/regulation-fcas-contribution-factors-procedure.pdf.
57. Ancillary services contribution factors. (2022). https://aemo.com.au/en/energy-systems/electricity/national-electricity-market-nem/system-operations/ancillary-services/ancillary-services-causer-pays-contribution-factors.
58. Global solar irradiance data and PV system power output data. (2019). https://solcast.com/.
59. Gannon, M. (2021). A comparison of large-scale solar farm revenue in 2020. https://wattclarity.com.au/articles/2021/03/gsd2020-a-comparison-of-large-scale-solar-farm-revenue-in-2020/.
60. Ancillary services payments and recovery. (2023). https://aemo.com.au/en/energy-systems/electricity/national-electricity-market-nem/data-nem/ancillary-services-data/ancillary-services-payments-and-recovery/.
61. Compliance Bulletin No 1 - Compliance with Dispatch Instructions, Offers and Bids. (2016) (Australian Energy Regulator (AER)). https://www.aer.gov.au/wholesale-markets/compliance-reporting/compliance-bulletin-no-1-compliance-with-dispatch-instructions-offers-and-bids.
62. NEM Registration Guide. (2021) (Australian Energy Market Operator (AEMO)). https://aemo.com.au/-/media/files/electricity/nem/participant_information/registration/generator/nem-generator-registration-guide.pdf.
63. Heptonstall, P.J., and Gross, R.J.K. (2020). A systematic review of the costs and impacts of integrating variable renewables into power grids. Nat. Energy *6*, 72–83.
64. Savelli, I., Hardy, J., Hepburn, C., and Morstyn, T. (2022). Putting wind and solar in their place: internalising congestion and other system-wide costs with enhanced contracts for difference in Great Britain. Energy Econ. *113*, 106218.
65. Rooftop solar helps send south Australia grid to zero demand in world first. (2021). https://reneweconomy.com.au/rooftop-solar-helps-send-south-australia-grid-to-zero-demand-in-world-first/.
66. Small-scale Renewable Energy Scheme. (2018) (Australian Government Clean Energy Regulator). http://www.cleanenergyregulator.gov.au/RET/About-the-Renewable-Energy-Target/How-the-scheme-works/Small-scale-Renewable-Energy-Scheme.
67. Leading the green economy. (2021). https://www.energymining.sa.gov.au/growth_and_low_carbon/leading_the_green_economy.
68. Solar feed-in payments. (2022). https://www.sa.gov.au/topics/energy-and-environment/energy-bills/solar-feed-in-payments.
69. Renewable Integration Study: Stage 1 Report. (2020) (Australian Energy Market Operator (AEMO)). https://aemo.com.au/-/media/files/major-publications/ris/2020/renewable-integration-study-stage-1.pdf.
70. AER approves costs for project EnergyConnect. (2021). https://www.aer.gov.au/news-release/aer-approves-costs-for-project-energyconnect.
71. Karimi-Arpanahi, S., and Pourmousavi, S.A. (2022). Efficient anomaly detection method for rooftop PV systems using big data and permutation

entropy. In 32nd Australasian Universities Power Engineering Conference (AUPEC) https://arxiv.org/abs/2301.06035.

72. Karimi-Arpanahi, S. (2023). Quantifying the predictability of renewable energy data for improving power systems decision making (v1.0). Preprint at Zenodo. https://doi.org/10.5281/zenodo.7538884.
73. Australian hourly solar irradiance gridded data. (2015). http://www.bom.gov.au/climate/how/IDCJAD0111.shtml.
74. Politi, A. (2017). Quantifying the dynamical complexity of chaotic time series. Phys. Rev. Lett. *118*, 144101.
75. Zunino, L., Zanin, M., Tabak, B.M., Pérez, D.G., and Rosso, O.A. (2010). Complexity-entropy causality plane: a useful approach to quantify the stock market inefficiency. Phys. Stat. Mech. Appl. *389*, 1891–1901.
76. Inouye, T., Shinosaki, K., Sakamoto, H., Toi, S., Ukai, S., Iyama, A., Katsuda, Y., and Hirano, M. (1991). Quantification of EEG irregularity by use of the entropy of the power spectrum. Electroencephalogr. Clin. Neurophysiol. *79*, 204–210.
77. Segal, M.R. (2004). Machine Learning Benchmarks and Random Forest Regression (Center for Bioinformatics and Molecular Biostatistics).
78. Quarterly Energy Dynamics Q4. (2020) (Australian Energy Market Operator (AEMO) (2021d)). https://aemo.com.au/-/media/files/major-publications/qed/2020/qed-q4-2020.pdf.
79. OpenNEM. (2022). https://opennem.org.au/energy/nem/?range=all&interval=quarter.
80. Mapping Australian photovoltaic installations. (2022). https://pv-map.apvi.org.au/historical.
81. Rostaghi, M., Ashory, M.R., and Azami, H. (2019). Application of dispersion entropy to status characterization of rotary machines. J. Sound Vib. *438*, 291–308.
82. Azami, H., Fernández, A., and Escudero, J. (2019). Multivariate multiscale dispersion entropy of biomedical times series. Entropy *21*, 913.
83. Li, X., Cui, S., and Voss, L.J. (2008). Using permutation entropy to measure the electroencephalographic effects of sevoflurane. Anesthesiology *109*, 448–456.
84. Zunino, L., Tabak, B.M., Serinaldi, F., Zanin, M., Pérez, D.G., and Rosso, O.A. (2011). Commodity predictability analysis with a permutation information theory approach. Phys. Stat. Mech. Appl. *390*, 876–890.
85. Kaffashi, F., Foglyano, R., Wilson, C.G., and Loparo, K.A. (2008). The effect of time delay on approximate & sample entropy calculations. Phys. Nonlinear Phenom. *237*, 3069–3074.
86. Li, X., Ouyang, G., and Richards, D.A. (2007). Predictability analysis of absence seizures with permutation entropy. Epilepsy Res. *77*, 70–74.
87. Yentes, J.M., Hunt, N., Schmid, K.K., Kaipust, J.P., McGrath, D., and Stergiou, N. (2013). The appropriate use of approximate entropy and sample entropy with short data sets. Ann. Biomed. Eng. *41*, 349–365.
88. Goerg, G. (2013). Forecastable component analysis. In International Conference on Machine Learning (PMLR), pp. 64–72.
89. Li, X., Li, D., Liang, Z., Voss, L.J., and Sleigh, J.W. (2008). Analysis of depth of anesthesia with Hilbert–Huang spectral entropy. Clin. Neurophysiol. *119*, 2465–2475.

Patterns, Volume *4*

Supplemental information

Quantifying the predictability of renewable energy data for improving power systems decision-making

Sahand Karimi-Arpanahi, S. Ali Pourmousavi, and Nariman Mahdavi

Note S1: Why is the predictability of PV generation not coupled with the factors currently being considered in the decision-making processes?

A crucial question regarding the importance of the proposed predictability measure for renewable generation is what additional information it can provide that current measures lack in the decision-making processes. In this respect, as the “fuel” of renewable energy sources is weather-related (e.g., solar irradiance or wind speed), a common counterargument is that the predictability of renewable energy sources is coupled with weather-related factors, which are already being considered.

To address this concern, we first acknowledge that PV generation predictability is coupled with short-term weather patterns (e.g., 1, 5 or 10-minute changes) since they are the main reason behind short-term changes in renewable generation. However, the way the weather-related factors are considered in decision-making processes does not bring into the picture the impact of these short-term changes on the predictability of renewable generation and its impacts; hence, these factors are not inherently correlated with the short-term predictability of renewable energy sources. To better explain the problem, let us focus on finding the best location for a solar farm, which typically involves the most comprehensive weather-related studies. Because of its impact on the return on investment, a detailed solar resource assessment is done by the investors to find the best location. Usually, they use earlier typical meteorological year (TMY) and satellite solar irradiance data to find one or more potential locations that would provide the highest annual yield. Then, a good practice is to install weather monitoring stations at potential locations to calibrate the satellite data and track the weather conditions over a period to improve the accuracy of yield estimation. Based on the current practices, the best location is the one that provides a high energy yield with minimal changes over the years and facilitates the safe operation of the solar farm. The former is estimated based on the solar irradiance metrics, such as monthly or annual global horizontal irradiance (GHI), direct normal irradiance (DNI), or global tilted irradiance (GTI), together with the cloud opacity or Clearness Index, in the potential solar farm locations. The safe operation is ensured based on tracking higher resolution meteorological data, such as wind gusts, rainfall, and clouds, to ensure the plant is safe from severe weather issues (such as floods), and it can operate safely during various events (for example during start-ups, shutdowns, and transients) [S1]. Therefore, if the safe operation of a plant can be ensured, the location with the highest solar power yield is typically chosen.

While affecting the decisions, none of these factors is inherently coupled with the short-term predictability of solar PV generation. Yet, the power grid’s safe and efficient operation depends on the predictability of net demand and generation. That is why current (and especially the future) electricity markets reward the generation with higher predictability.

Here, we intend to reject a potential counterargument for the importance of PV generation predictability: the proposed predictability measure (WPE of dimension 6 and a

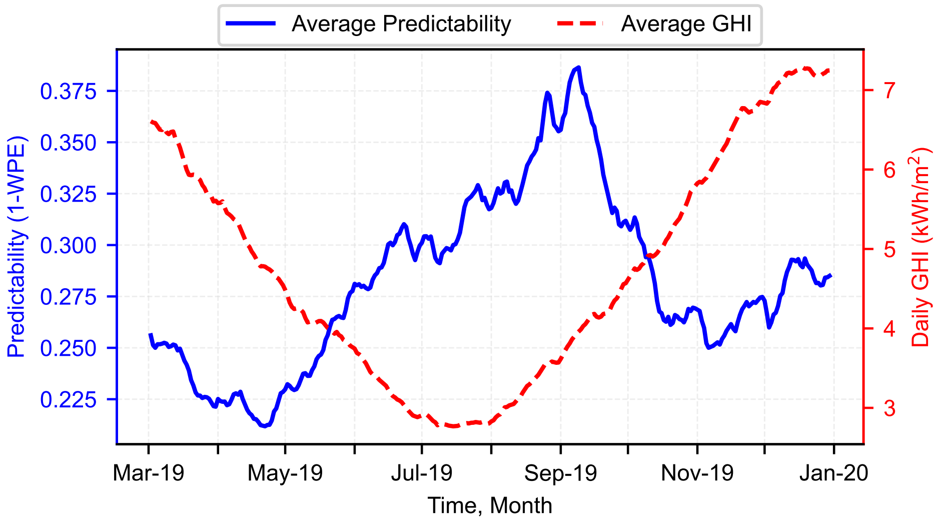

Figure S1. Negative correlation between the predictability of PV generation and the average daily GHI over time in NSW.

resampling interval of 10 minutes) is correlated with the monthly or annual solar irradiance. In other words, a location with higher all-sky solar irradiance (i.e., solar irradiance when the cloud opacity and its impact on the irradiance at the land surface is considered) would have higher generation predictability. As annual or monthly solar irradiance is the most critical factor in such decisions, if there was a link between the two, considering predictability would become unnecessary. Therefore, we have conducted the following analyses to reject this hypothesis.

Using our real-world PV generation dataset [S2], we first demonstrate that the predictability of PV generation is not coupled with solar irradiance. To do so, we compare the PV generation predictability (1-WPE) of all rooftop systems in the states of South Australia (SA) and New South Wales (NSW) over a year (by rolling two-month windows) with the average all-sky GHI in the same locations. Our rooftop PV generation dataset, respectively, includes the time series of 106 and 74 postcodes in the two states. Also, GHI data of these postcodes were obtained from the National Aeronautics and Space Administration (NASA) Langley Research Center (LaRC) Prediction of Worldwide Energy Resource (POWER) Project funded through the NASA Earth Science/Applied Science Program [S3]. Figures S1 and S2 show the average PV generation predictability and the average daily GHI over time for the states of NSW and SA, respectively. In SA, there is a positive correlation between the average GHI and the predictability over time, while in NSW, there is a negative correlation. In other words, unlike in SA, the two parameters not only show no meaningful correlation in NSW but also are negatively correlated. This means that although they can be correlated over time in some locations

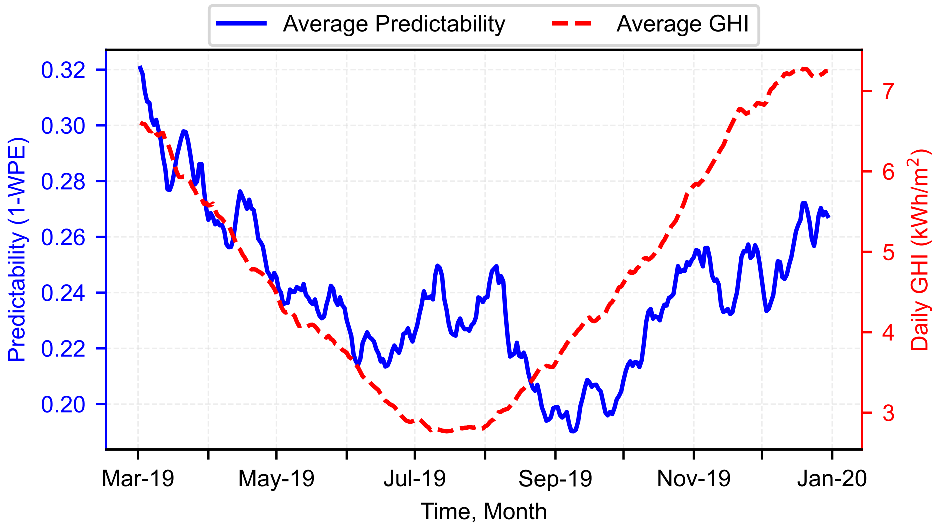

Figure S2. Positive correlation between the predictability of PV generation and the average daily GHI over time in SA.

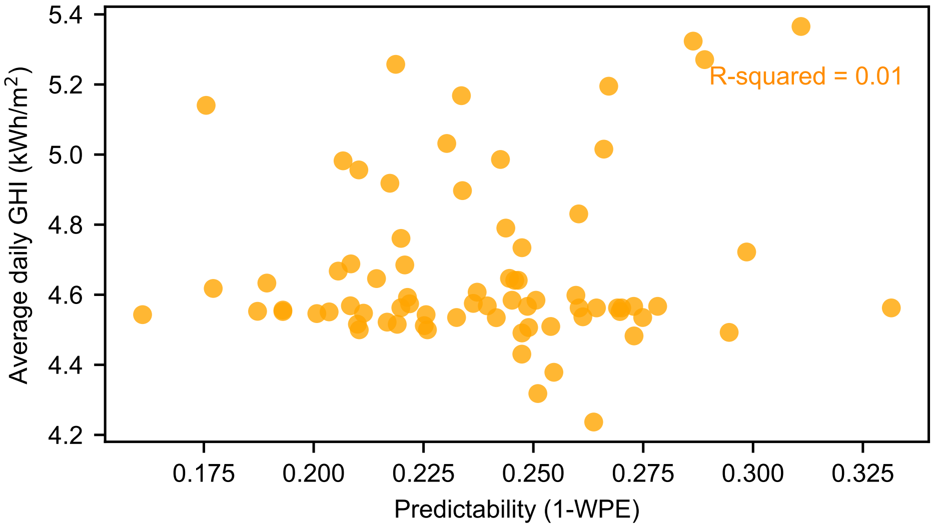

Figure S3. No correlation between the predictability of PV generation and the average daily GHI in different postcodes of NSW.

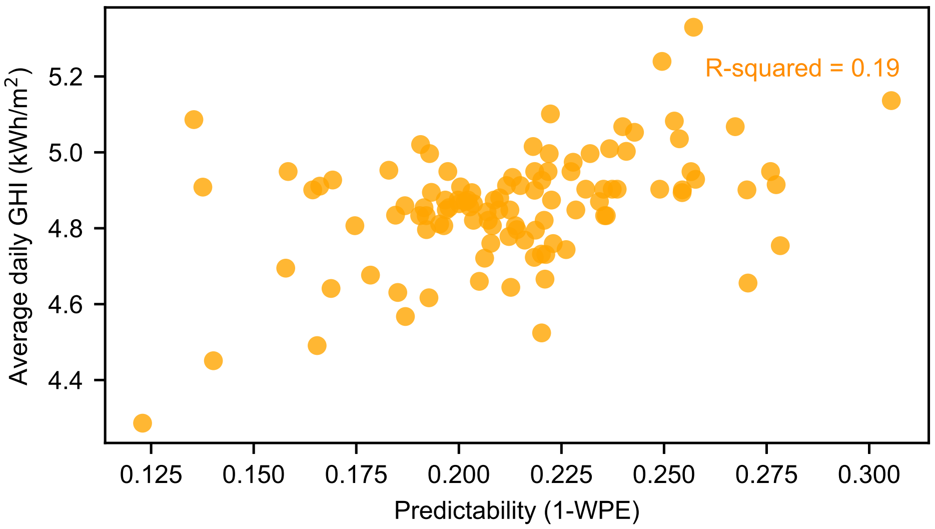

Figure S4. **Weak positive correlation between the predictability of PV generation and the average daily GHI in different postcodes of SA.**

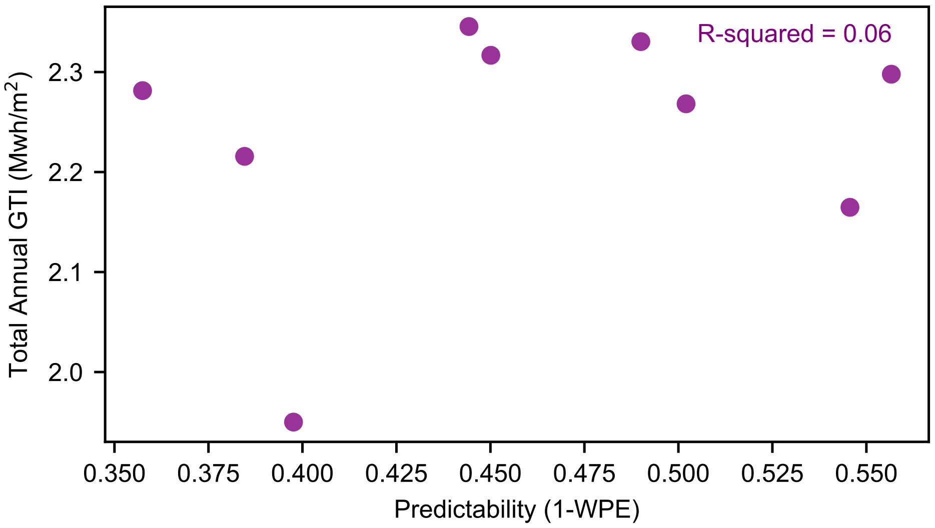

Figure S5. **No correlation between the predictability of GTI and the annual GTI in potential solar farm locations in NSW.**

(due to the weather patterns of that region), we cannot generalize the existence of the correlation to every region. This rejects the assumption that all-sky solar irradiance (or other associated factors) and PV generation predictability are inherently coupled.

Furthermore, to test the existence of any coupling between the PV generation predictability and the average daily solar irradiance of different locations, we calculated the correlation between these two factors for different postcodes in SA and in NSW, using our rooftop PV generation dataset and GHI data obtained from [S4]. As shown in Figure S3, there is no meaningful correlation between these two factors in NSW. However, doing the same analysis for SA, we found a weak correlation between these two factors, as shown in Figure S4. As previously noted, while there might be a correlation between these two factors in some locations, it is not universal; hence, they are not inherently coupled, and one cannot be used as a proxy variable for the other in decision-making processes.

Additionally, to check if the independence between predictability and average solar irradiance holds for solar farm locations in NSW (which usually have relatively high irradiance compared to urban areas), we used a dataset consisting of the 5-minute GTI time series (from August 2021 to August 2022) for single-axis sun-tracking panels in 9 potential solar farm locations in NSW. Figures 8.(a) and 8.(b) in the main manuscript, respectively, show the total annual GTI values and the predictability for these locations in NSW. We can see that the locations with higher annual GTI do not necessarily have higher predictability. For better illustration, Figure S5 shows the scatter plot of two parameters, exhibiting no correlation between them. This analysis shows that the short-term predictability of GTI (or solar irradiance) in a location is not coupled with its monthly or annual GTI, similar to the PV generation. Consequently, the location with the highest solar irradiance might not necessarily present high predictability of PV generation.

References

S1. Mehos, M. *et al.* Concentrating solar power best practices study. Tech. rep. (National Renewable Energy Lab.(NREL), Golden, CO (United States), 2020).

S2. 2019 Australian Voltage and PV Generation as part of ARENA grant ARP128. `http://www.solaranalytics.com/data`.

S3. The Prediction of Worldwide Energy Resources (POWER) Project. 2022. `https://power.larc.nasa.gov/`.

S4. Australian Hourly Solar Irradiance Gridded Data. The Bureau of Meteorology, 2015. `http://www.bom.gov.au/climate/how/IDCJAD0111.shtml`.
